# Deep-learning methods for contrast enhancement and artifact reduction in cryo-electron tomography: a systematic analysis of the state of the art and proposed improvements

**DOI:** 10.1107/S2059798326001166

**Published:** 2026-02-19

**Authors:** Henry N. Jones, Aneesh Deshmukh, Kanupriya Pande

**Affiliations:** a Molecular Biophysics and Integrated Bioimaging, Lawrence Berkeley National Laboratory, Berkeley, California, USA; b Center for Applied Mathematics for Energy Research Applications, Lawrence Berkeley National Laboratory, Berkeley, California, USA; c Department of Chemistry, Bridge Institute, Michelson Center for Convergent Bioscience, University of Southern California, Los Angeles, California, USA; National Center of Biotechnology, CSIC, Spain

**Keywords:** deep learning, cryo-electron tomography, contrast enhancement, missing wedge

## Abstract

Cryo-electron tomography (cryo-ET) has emerged as the preferred technique for visualizing the organization of macromolecular complexes *in situ* and resolving their structures at subnanometre resolution [Tegunov *et al.* (2021) ▸, *Nat. Methods*, **18**, 186–193]. Despite improvements in data quality as a result of advances in detector technology, microscope stability and stage precision, the analysis and interpretation of tomograms remains challenging due to a low signal-to-noise ratio and reconstruction artifacts stemming from experimental constraints in specimen tilt during data collection resulting in a missing wedge in the Fourier space. Recently, self-supervised deep-learning methods have been proposed for contrast enhancement and reduction of resolution anisotropy in reconstructed tomograms. Here, we evaluate several state-of-the-art deep-learning methods which aim to improve the interpretability of cryo-ET reconstructions, with a focus on their performance on downstream tasks of template matching, sub­tomogram averaging and segmentation. We propose new training architectures and a loss function based on Fourier shell correlation that show improved performance over the standard U-Net with L_1_/L_2_ losses. We demonstrate our analysis on four diverse experimental datasets: purified 80S ribosomes, *in situ Chlamydomonas reinhardtii*, immature HIV-1 virus-like particles and INS-1E cells.

## Abstract 2

Cryo-ET is a rapidly emerging technique that enables 3D visualization of complex biological structures, but present limits on signal-to-noise ratio and reconstruction quality pose challenges for downstream analysis. Here, we present a systematic analysis of state-of-the-art deep-learning methods for contrast enhancement and propose improvements in neural network architectures and training objectives to preserve more high-resolution information.

## 1. Introduction

Cryo-electron tomography (cryo-ET) is a powerful technique for imaging the organization and three-dimensional (3D) structure of macromolecular complexes in their native cellular environment. The technique works by reconstructing a 3D volume, called a tomogram, of a cellular sample from two-dimensional (2D) projections collected as the sample is rotated about an axis. In most cases, the tilt series is aligned by tracking fiducial markers or strong cellular features, and reconstructed using filtered back-projection (FBP). This is followed by downstream tasks such as template matching (TM) for picking subtomograms of target molecules, and iterative subtomogram alignment (STA) and averaging for improving the resolution and signal-to-noise ratio (SNR) of the molecular densities (Wan *et al.*, 2024 ▸). Another important application is the annotation and segmentation of cellular structures such as organelles, membranes and macromolecular complexes to visualize the ‘molecular sociology’ of the cell (Robinson *et al.*, 2007 ▸; Chen *et al.*, 2017 ▸). In practice, downstream data analyses on 3D tomograms are often limited due to fundamental challenges of the data-acquisition and reconstruction processes.

The first challenge is the extremely poor signal-to-noise ratio of cryo-ET tilt series as a result of low electron dose during imaging to limit radiation damage to sensitive biological structures. The second challenge is the appearance of anisotropic reconstruction artifacts due to the missing wedge of information in Fourier space that arises because the range of projection angles is limited due to experimental constraints, usually to ±60° rather than the desired full range of ±90°. Locating target molecules in crowded cellular environments with high confidence by either manual or automated picking algorithms, and accurately extracting and aligning subtomograms, is dependent on the quality of the reconstructed tomograms. Consequently, there has been a considerable focus on the development of computational approaches for contrast enhancement of low-SNR tomograms and the correction of missing-wedge artifacts.

Traditional methods for improving contrast in cryo-ET volumes reconstructed with filtered back-projection (FBP) involve spatial down-sampling, low-pass filtering and deconvolution with a Wiener filter. On the other hand, reconstruction methods such as the simultaneous iterative reconstruction technique (SIRT; Gilbert, 1972 ▸) and model-based iterative reconstruction (MBIR; Yan *et al.*, 2019 ▸) iteratively increase the consistency between measured and calculated reprojections of the tomogram and may employ hand-crafted priors (*e.g.* positivity and compressed sensing; Leary *et al.*, 2013 ▸; Deng *et al.*, 2016 ▸) as constraints, which have been shown to reduce the missing-wedge artifacts to varying degrees. More recently, motivated by the success of deep neural networks in computer vision tasks such as image segmentation, classification and missing-data imputations, convolutional neural network (CNN)-based methods have been proposed for cryo-ET denoising and missing-wedge compensation.

### 1.1. Deep learning for denoising and missing-wedge compensation

Image denoising is the process of estimating signal from a corrupted or incomplete measurement. If *x* ∈ *X* = *R*^*m*^ and *y* ∈ *Y* = *R*^*m*^ denote clean and noisy images, respectively, then the measurement can be described as 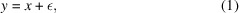
where ε ∈ *R*^*m*^ is the unknown noise vector. Traditional denoising techniques assume *a priori* knowledge of signal and noise statistics. When large volumes of training data are available, instead of relying on explicit image priors, deep CNNs have been shown to be versatile at learning the mapping *f*_θ_ from corrupted measurements to unobserved clean signal by training a regression model under a loss function *d*(*y*, *x*), 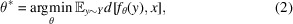
where θ are the weights of the neural network and 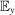 is the expected prediction error over *y*. In supervised training paired ground-truth images *x* are available for the noisy measurements *y*. Although this approach has been shown to provide significantly improved performance compared with classical denoising algorithms (Dabov *et al.*, 2007 ▸), in real-world applications the acquisition of clean paired measurements is difficult or even impossible. To address this issue, denoisers that can be trained without clean paired data have been proposed, including *Noise*2*Noise *(N2N; Lehtinen *et al.*, 2018 ▸), *Noise*2*Void* (N2V; Krull *et al.*, 2019 ▸) and *Noise*2*Self* (N2S; Batson & Royer, 2019 ▸), where the target image *y*′ is related to the noisy measurement *y* in unique ways. N2N training may be applied if it is possible to measure independent noisy instances *y*′ of the same underlying signal *y*. N2V generates target *y*′ by applying a blind spot to input *y*, while in N2S a *J*-invariant mask is applied to the image to generate input *y* and the target *y*′ is generated by applying the complementary mask to ensure that the loss compares two statistically independent images.

The goal of denoising in cryo-ET is to improve the contrast (*i.e.* signal-to-noise ratio) of the 3D tomogram **x** ∈ *R^n^* reconstructed from the noisy 2D projections **y** ∈ *R^m^*,
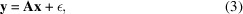
where the linear forward operator **A** : *R^n^* → *R^m^* describes the measurement process and the noise is modeled as an additive term, but in general can be non-additive; for example, Poisson noise, where the noise term depends on the measured signal (Kang *et al.*, 2018 ▸). The most common approach for reconstruction is the filtered back-projection (FBP) algorithm, which applies a linear back-projection operator **A**^†^ : *R^n^* → *R^m^* that takes the measurements into the reconstruction space, given as 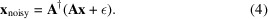
Application of the back-projection operator results in spatially correlated noise in the reconstructed volume, even if the measurement noise ε is element-wise independent in the projection space. Additionally, direct reconstruction methods such as FBP give rise to pronounced missing-wedge artifacts manifested as angular streaking and elongation of features along the *Z* axis (Moebel & Kervrann, 2020 ▸). Simply denoising the projection images prior to reconstruction disturbs the linearity between measurements and reconstruction and produces artifacts in the reconstruction, as discussed in Buchholz *et al.* (2019 ▸). At the same time, due to the voxel-wise statistical dependence in reconstruction space, cryo-ET reconstructions cannot be denoised using training pairs generated from real-space voxel samplings, as is possible for nontomographic volumes (*e.g.Neighbor*2*Neighbor*; Huang *et al.*, 2021 ▸). A better approach is to train a denoising network on input–target pairs created from 3D reconstructions of data partitioned in the projection space, where the noise can be assumed to be statistically independent.

*CryoCare* (Buchholz *et al.*, 2019 ▸) is the initial framework for N2N training to denoise cryo-electron microscopy (cryo-EM) and cryo-ET data. While it is impossible to measure two noisy instances of the same sample during cryo-ET data acquisition due to the effects of radiation damage, training data with independent noise may be generated by (i) splitting the acquired tilt series into two halves with even and odd tilts (T2T) or (ii) splitting the dose-fractionated movie frames for each tilt into two halves with motion-corrected and averaged even and odd frames (F2F). Both of the above strategies ultimately result in tomograms with additional noise due to reducing the amount of signal in the tilt images that are used for reconstruction (Buchholz *et al.*, 2019 ▸; Peck *et al.*, 2025 ▸). In the case of T2T, the loss of signal is in the form of increased angular increment between successive tilts, while in the case of F2F the signal in each tilt image is effectively halved due to averaging over a half-set of the movie frames. After reconstructing independent tomograms from the data halves, noisy input–target training pairs are created by extracting sub­volumes from the two tomograms. *Topaz-denoise* (Bepler *et al.*, 2020 ▸) adapts the *CryoCare* N2N framework to train a general denoising model (pre-trained model) on a large cryo-ET data set from diverse samples and across a variety of imaging conditions. *IsoNet*, a deep-learning method based on the *Noisier*2*Noise* (Moran *et al.*, 2020 ▸) framework for compensating missing-wedge information, works without the need to split tilt-series data into two halves (Liu *et al.*, 2022 ▸). Since cryo-ET volumes contain multiple copies of the same macromolecule in random orientations, *IsoNet* exploits the redundancy of information to find the mapping from input subtomograms corrupted with an additional artificial missing wedge and target subtomograms that only have the original missing wedge. While native *IsoNet* is designed to iteratively predict information in the missing wedge, it may be trained to jointly denoise by adding noise onto the subtomograms that are not corrupted with the additional missing wedge; however, this requires manual hyperparameter tuning of the noise-addition schedule. *DeepDeWedge* (Wiedemann & Heckel, 2024 ▸) extends the self-supervised N2N denoising framework to also jointly learn the frequencies in the missing wedge by augmenting the training data with randomly rotated extracted subtomograms and further corrupting the input with an artificial missing wedge. As discussed in detail in Section 3.2.1, a drawback of N2N-like approaches is that they enable increased image contrast by suppressing high-frequency information. Recently, *CryoSamba* (Costa-Filho *et al.*, 2025 ▸) has been proposed as a deep-learning approach that improves contrast by interpolating between neighboring slices using an optical flow-based neural network with less aggressive suppression of high-frequency structural information.

Here, we evaluate the state-of-the-art deep-learning methods for improving the quality of noisy 3D tomograms and demonstrate their performance on four diverse data sets. Of primary interest is high-resolution denoising at relatively low binning levels/small pixel size to help elucidate sparsely populated, pleomorphic molecular complexes for which it is difficult or impossible to obtain high-resolution averages due to their relative sparsity. To this end, we incorporate several recent, attention-enabled neural network architectures and a frequency-space loss based on the Fourier shell correlation (FSC) to examine directions for the improvement of high-resolution image restoration for cryo-ET. We assess existing and proposed methods on a variety of data sets through qualitative comparison of real-space and Fourier-space slices, quantitative SNR metrics, TM, STA and segmentation. We ultimately want to know the limits of self-supervised image restoration for biology, and if a hard tradeoff between reliable high-frequency information and interpretability exists, what utility self-supervised image-restoration methods serve.

## 2. Methods

### 2.1. Cryo-ET data sets

#### 2.1.1. Purified *Saccharomyces cerevisiae* 80S ribosomes

The first data set was obtained from the EMPIAR database (Iudin *et al.*, 2022 ▸) deposited under accession number EMPIAR-10045 and contains seven tilt series collected on a sample of purified 80S ribosome from *S. cerevisiae* (Bharat & Scheres, 2016 ▸). This data set is commonly used for benchmarking algorithms for cryo-ET and consists of aligned tilts and reconstructed tomograms.

#### 2.1.2. Cryo-plasma FIB-milled *Chlamydomonas reinhardtii*

The second data set is from *C. reinhardtii*, a model organism for the *in situ* visualization of numerous fundamental cellular processes, deposited with accession code EMPIAR-11830 (Kelley *et al.*, 2026 ▸). These *in situ* cryo-ET data were collected on samples of *C. reinhardtii* after cryogenic plasma-based focused ion-beam (FIB) milling. To encompass a diversity of lamella thicknesses, apparent tomogram quality and biological structures, we selected tomograms 11, 16, 19 and 24 to assess the effect of denoising on the performance of membrane segmentation and provide a comparison of the predominant training pair-generation schemes (*i.e.* tilt angle and movie-frame splitting).

#### 2.1.3. INS-1E cells

The third data set is from an in-house pancreatic β-cell line. Tilt series were collected at the periphery of the cell using *Tomo* 5.18.0 at angles ranging from −60° to +60° in a dose-symmetric fashion with 3° increments, with the defocus modulated between −4 and −6 µm. This data set was used to study the effect of denoising on the segmentation of a non-FIB-milled sample.

#### 2.1.4. Purified immature HIV-1 virus-like particles (VLPs)

To further study the performance of attention-enabled architectures, and the effect of denoising 3D TM, we selected the five-tomogram subset (tomograms 1, 3, 43, 45 and 54) of EMPIAR-10164 (Schur *et al.*, 2016 ▸) often used in benchmarking STA (Turoňová *et al.*, 2017 ▸; Scaramuzza & Castaño-Díez, 2021 ▸).

### 2.2. Cryo-ET data processing

#### 2.2.1. Tomogram reconstruction

We used *IMOD* (Kremer *et al.*, 1996 ▸) for our entire tomographic reconstruction pipeline. For INS-1E cells and HIV-1 VLP tomograms, processing began with motion correction using *MotionCor*3 (Zheng *et al.*, 2017 ▸). We used deposited aligned tilt series for EMPIAR-10045 and EMPIAR-11830. We primarily used the default *IMOD* parameters for cryo-ET reconstructions as detailed in the *IMOD* tomography guide (Mastronarde, 2024 ▸), although we aimed to maintain high-frequency information below the Nyquist at all stages of tilt-series processing to assess the degree to which denoising removes or otherwise alters high-frequency structural details. We applied dose weighting and used a 15 nm defocus step for 3D contrast transfer function (CTF) correction (Turoňová *et al.*, 2017 ▸). We super-sampled both input and reconstruction by a factor of three to limit artifacts in Fourier space. During reconstruction and subsequent downsampling, tomograms were low-pass filtered using *reducefiltvol* in *IMOD* with cutoff and Gaussian roll-off of 0.42 and 0.03 cycles per pixel to suppress signal beyond the Nyquist. To assess the denoising performance using the full available signal, EMPIAR-10164 tomograms were Fourier binned to prevent anti-aliasing without additional low-pass filtering. To fairly assess missing-wedge compensating tools such as *IsoNet* and *DeepDeWedge*, we only selected tilt series with an approximately 60° missing wedge, so we omitted tomogram 11 from EMPIAR-10045. Training tomograms for INS-1E and EMPIAR-10045 were split with the T2T scheme, while EMPIAR-10164 tomograms were split with the F2F scheme. Except for the comparison of splitting schemes in Supplementary Fig. S5, denoising on EMPIAR-11830 *Chlamydomonas* tomograms was performed with the F2F scheme. Both movie-frame split (F2F) and tilt-angle split (T2T) tomograms were reconstructed using the alignment and CTF parameters computed from the full reconstruction.

#### 2.2.2. Template matching

3D TM on EMPIAR-10045 was performed using *GAPSTOP-TM* (Cruz-León *et al.*, 2024 ▸) at the 4× binning level (8.704 Å per voxel) using the map originally deposited with this data set as a template (EMDB entry EMD-3228; Bharat & Scheres, 2016 ▸). The authors of *GAPSTOP-TM* showed that TM detection benefits from retaining high-resolution information in the template (although at the risk of introducing template bias). Therefore, to investigate the effect of the ability of the denoising method to retain high-frequency structural details on TM detection, and to compare with the optimal TM results on raw data, we did not reduce the resolution of the 4× binned template with additional low-pass filtering. We used a soft mask contoured to the template with Gaussian attenuation with a 10° angular search. Observing significant differences in score distribution means and standard deviations between raw and denoised score map distributions (see Supplementary Fig. S8), we extracted particles with TM scores above the 99.5th percentile in order to fairly assess the stability of STA orientations and quality of picked particles through our STA analyses.

We similarly performed 3D TM on EMPIAR-10164 using *GAPSTOP-TM* at the 4× binning level (5.4 Å per voxel) using EMDB entry EMD-16207 as a template (Zivanov *et al.*, 2022 ▸). Similar to the ribosome TM, we did not low-pass filter the template. We used a soft contoured mask with a 10° angular search and *C*6 symmetry. To focus our TM analysis to regions away from beads and therefore simplify our STA procedure, we manually masked beads in the volumes prior to particle picking. For the establishment of high-fidelity reference STA parameters for the raw data, we found thresholding at the ∼99.96th percentile to give the ∼20 000 particles expected in the literature (Burt *et al.*, 2021 ▸; Wan *et al.*, 2024 ▸).

To quantify the effect of denoising on particle selection, we calculated precision, recall and the area under the precision–recall curve (PR-AUC) between found positions and a curated ground-truth list (Xu *et al.*, 2011 ▸; Langlois *et al.*, 2011 ▸). A found peak was considered to be a match if the peak position was within a set tolerance distance from a ground-truth position. If the number of true positives (correctly predicted positive particles), false positives (incorrectly predicted positive particles) and false negatives (actual positive particles incorrectly predicted as negative) is *n*_tp_, *n*_fp_ and *n*_fn_, respectively, then precision, recall and F1 score are defined as 
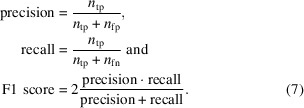
The precision and recall values are calculated for different thresholds of the cross-correlation (CC) map, and PR-AUC is computed as the area under precision–recall curve, where precision is on the *y* axis and recall is on the *x* axis.

#### 2.2.3. Subtomogram averaging

STA was performed using *STOPGAP* (Wan *et al.*, 2024 ▸) guided by the tutorial provided by the authors (S. J. Rothfuss & W. Wan; https://subtomo.readthedocs.io/hiv/bin4/). For the EMPIAR-10045 tomograms, particles were extracted from 4× binned tomograms with a box size of 50 × 50 × 50. We performed two iterations of alignment with 5° angular increment and three angular iterations, followed by two iterations with 3° angular increment and three angular iterations; these iterations had low-pass filter cutoffs of 12, 14, 16 and 18 Fourier pixels, respectively. We then rescaled the 4× binned particle list to the 2× binning level. We then performed six iterations of alignment: two iterations with 3° angular increment and four angular iterations, two iterations with 2° angular increment and three angular iterations, and two iterations with 1.5° angular increment and two angular iterations; these iterations had low-pass filter cutoffs of 24, 28, 32, 36, 40 and 44, respectively. We performed a final iteration with 1° angular increment, two angular iterations and a low-pass filter cutoff of 46 Fourier pixels. All iterations used a spherical, binary cross-correlation mask with a five-pixel radius. After the first iteration of alignment at the 2× binning level, we created a soft mask contoured to the density which was used for all subsequent alignment iterations. Unless otherwise noted, it is with this mask that we report final FSC calculations, which appeared appropriate due to negligible difference between the uncorrected FSC and the *STOPGAP*-corrected FSC curve determined by phase randomization.

For EMPIAR-10164, STA was performed only on the raw data to create a curated ground truth for TM and resolution preservation analyses. The 4× binned TM particle list was rescaled to the 2× binning level and particles were extracted with a box size of 128 × 128 × 128. We performed four iterations of alignment: two iterations with a 4° angular increment and three angular iterations, and two iterations with a 2° angular increment and three angular iterations. These iterations had low-pass filter cutoffs of 30, 34, 38 and 42. All iterations used a spherical, binary cross-correlation mask with radius 10 and an elliptical real-space mask including the six capsids surrounding the central capsid. After the first alignment iteration, we observed a distinct number of low-scoring particles, which we cleaned with a CC threshold of 0.07, leaving 18 558 particles.

#### 2.2.4. Signal-to-noise quantification

We followed the method outlined in Bepler *et al.* (2020 ▸) to quantify the SNR of raw and denoised tomograms by selecting paired signal and background regions. Given *N* signal, background pairs 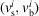, the SNR is given by 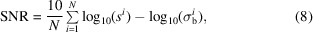
where 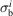 is the variance in background 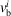, and 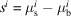, where 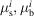 is the mean in signal, background indexed by *i*.

For EMPIAR-10064 and EMPIAR-10045, the amount of ice allowed us to use *IsoNet* masking functions on *CryoCare*-denoised volumes to create reliable sample and background masks. We used these masks to extract 300 sample and 300 background regions. For both data sets we used the STA box size at the corresponding binning level (50 × 50 × 50 for 4× binned EMPIAR-10045 and 64 × 64 × 64 for 4× binned EMPIAR-10164). The crowding of the INS-1E cells made an automated approach difficult, so we manually selected 30 cubes of sample and background (ice) with a box size of 32 × 32 × 32 for SNR estimation.

#### 2.2.5. Membrane segmentation

*TomoSegMemTV*, a contrast-based segmentation module (Martinez-Sanchez *et al.*, 2014 ▸), was used to automatically segment lipid membranes in INS-1E cells and FIB-milled *C. reinhardtii* data sets (Fig. 7) using surfaceness values of 0.1 and 0.025, respectively. Subsequent manual annotation of organelles and subcellular lipid membranes was performed using *Amira* (Thermo Fisher Scientific).

## 3. Results

### 3.1. Neural network architecture for better contrast enhancement

**Figure 1:**
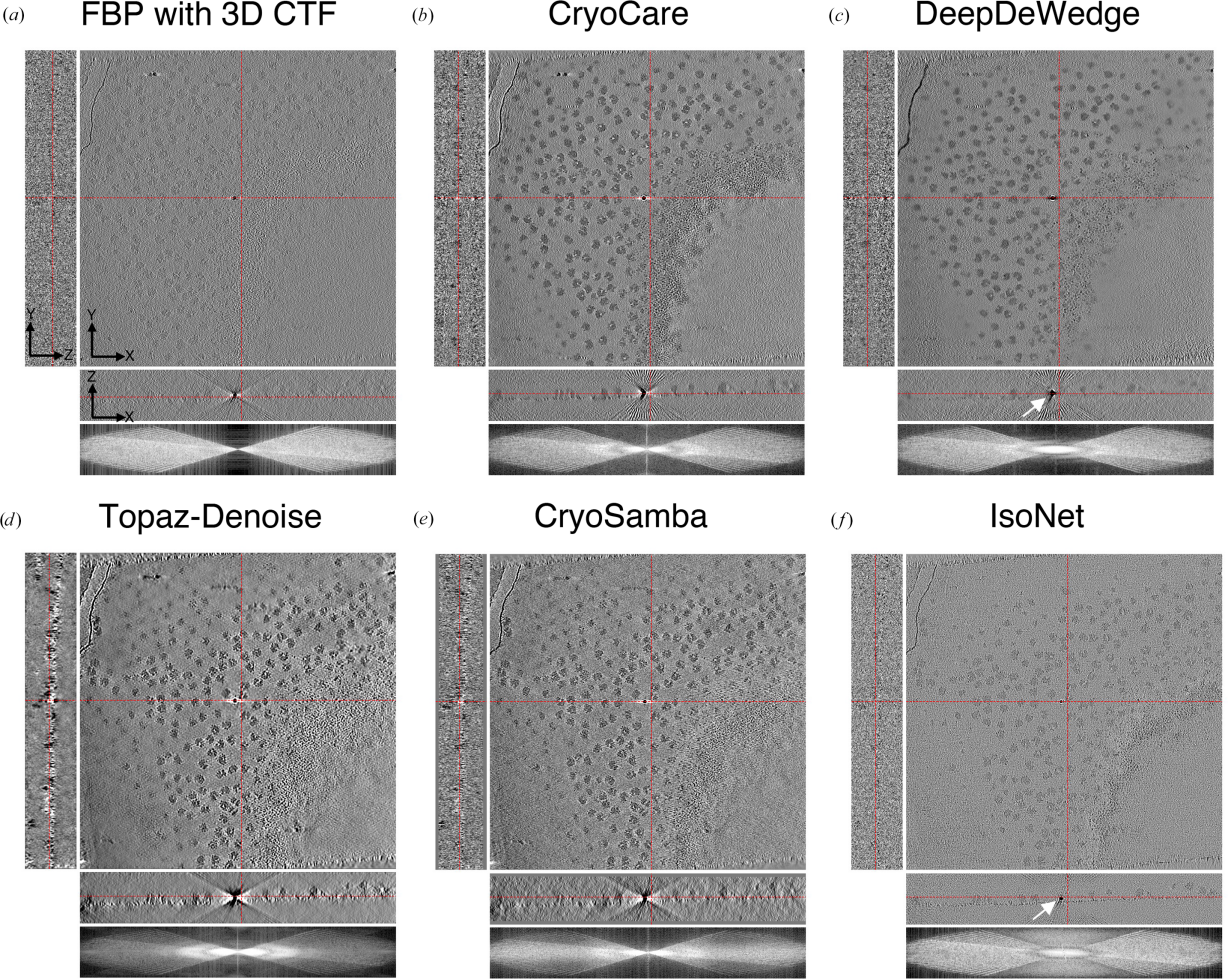
Visual comparison of deep-learning methods. Orthogonal slices through the tomogram and the log-magnitude Fourier space are shown for tomogram 5 of the EMPIAR-10045 data set at 4× binning for (*a*) the raw tomogram reconstructed with filtered back-projection and 3D CTF correction as implemented in *IMOD* and after denoising with (*b*) *CryoCare*, (*c*) *DeepDeWedge*, (*d*) *Topaz-denoise*, (*e*) *CryoSamba* and (*f*) *IsoNet*. *CryoCare*, *Topaz-denoise* and *CryoSamba* are denoising methods, while *DeepDeWedge* and *IsoNet* are methods for joint denoising and missing-wedge compensation. *IsoNet* is better at missing-wedge compensation, as can be seen by the appearance of filled-in frequencies in Fourier space.

In Fig. 1 ▸ we show a comparison of these methods for tilt-series data collected from purified *S. cerevisiae* 80S ribosome deposited in EMPIAR-10045 at the 4× binning (8.704 Å per voxel) level. *IsoNet* was trained with the default noise-addition scheme, while we used the pretrained, 10 Å denoising model provided by *Topaz* (Bepler *et al.*, 2020 ▸). All four N2N-like methods are effective in denoising the ribosome tomograms by smoothing the background while still preserving structural features. While both *IsoNet* and *DeepDeWedge* are able to compensate the frequencies in the missing wedge, as evident by the reduction in the streaky artifacts of the fiducial marker (white arrow on the *XZ* plane), it is clear from qualitative analysis of orthogonal slices that *DeepDeWedge* is more efficient at contrast enhancement, while *IsoNet* performs better in learning the missing frequencies, as shown by the log-magnitude Fourier space. *CryoSamba* appears to be less effective at contrast enhancement than N2N-like methods at this binning level, and while it is closer in denoising to *Topaz* and *CryoCare* in the *XY* plane, the *XZ* plane is much less interpretable than in all other methods.

The existing N2N-like approaches for denoising cryo-ET data (*CryoCare*, *DeepDeWedge*, *IsoNet* and *Topaz-denoise*) have employed a standard U-Net (Ronnenberger *et al.*, 2015 ▸) as the model architecture. A drawback of the U-Net architecture (that is based on CNNs) is the inability to learn global information and the significantly large number of trainable parameters (in the tens of millions for a simple three-layer U-Net) that necessitates large amounts of training data to achieve accurate results. Here, we demonstrate the performance of *CryoCare* and *DeepDeWedge* with two types of alternative architectures that have recently gained popularity in the computer vision community: the mixed-scale dense network (MS-D Net; Pelt & Sethian, 2018 ▸) and hybrid U-Net–Vision Transformer (ViT)-based architectures that adopt a 3D shifted-window (Swin) transformer. MS-D Net has been shown to achieve accurate results with relatively fewer training parameters compared with U-Net by using dilated convolutions instead of scaling operations to capture features at different image scales as well as dense connections between layers. The lower number of training parameters not only reduces the requirements on the amount of training data required for comparable performance, but also reduces the risk of overfitting to the noise. ViT-based architectures such as SwinUNETR (Tang *et al.*, 2022 ▸) and Swin-Conv U-Net (SCUNet; Zhang *et al.*, 2023 ▸) combine the advantages of residual convolutions for local modeling and Swin transformers for nonlocal modeling, and have recently been used to improve the quality of cryo-EM maps (He *et al.*, 2023 ▸). Further details of architectures are given in Section S1.

**Figure 2:**
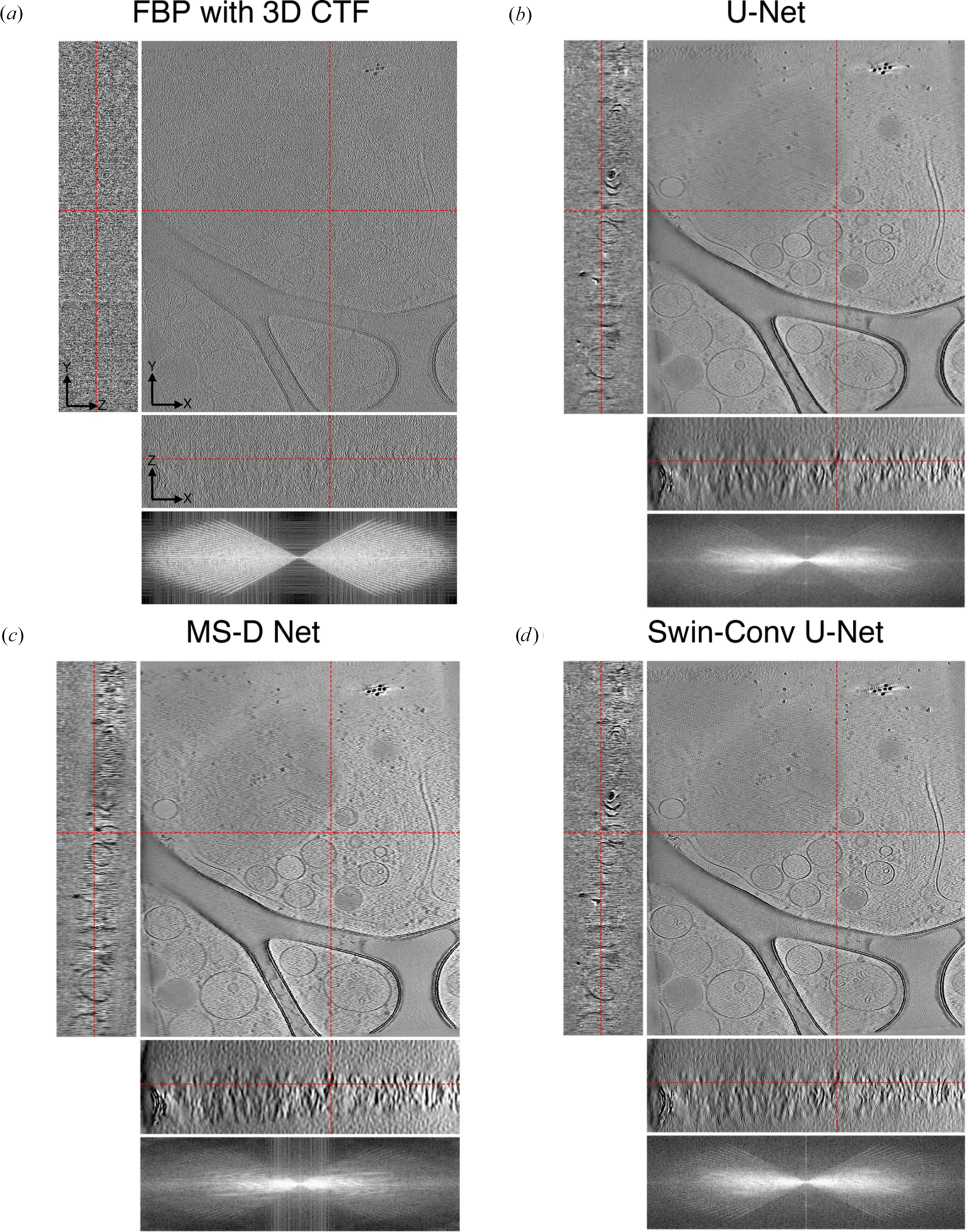
Visual comparison of (*a*) a raw tomogram reconstructed with FBP and 3D CTF correction and tomograms denoised by a trained *CryoCare* model based on (*b*) U-Net, (*c*) MS-D Net and (*d*) SCUNet.

**Figure 3:**
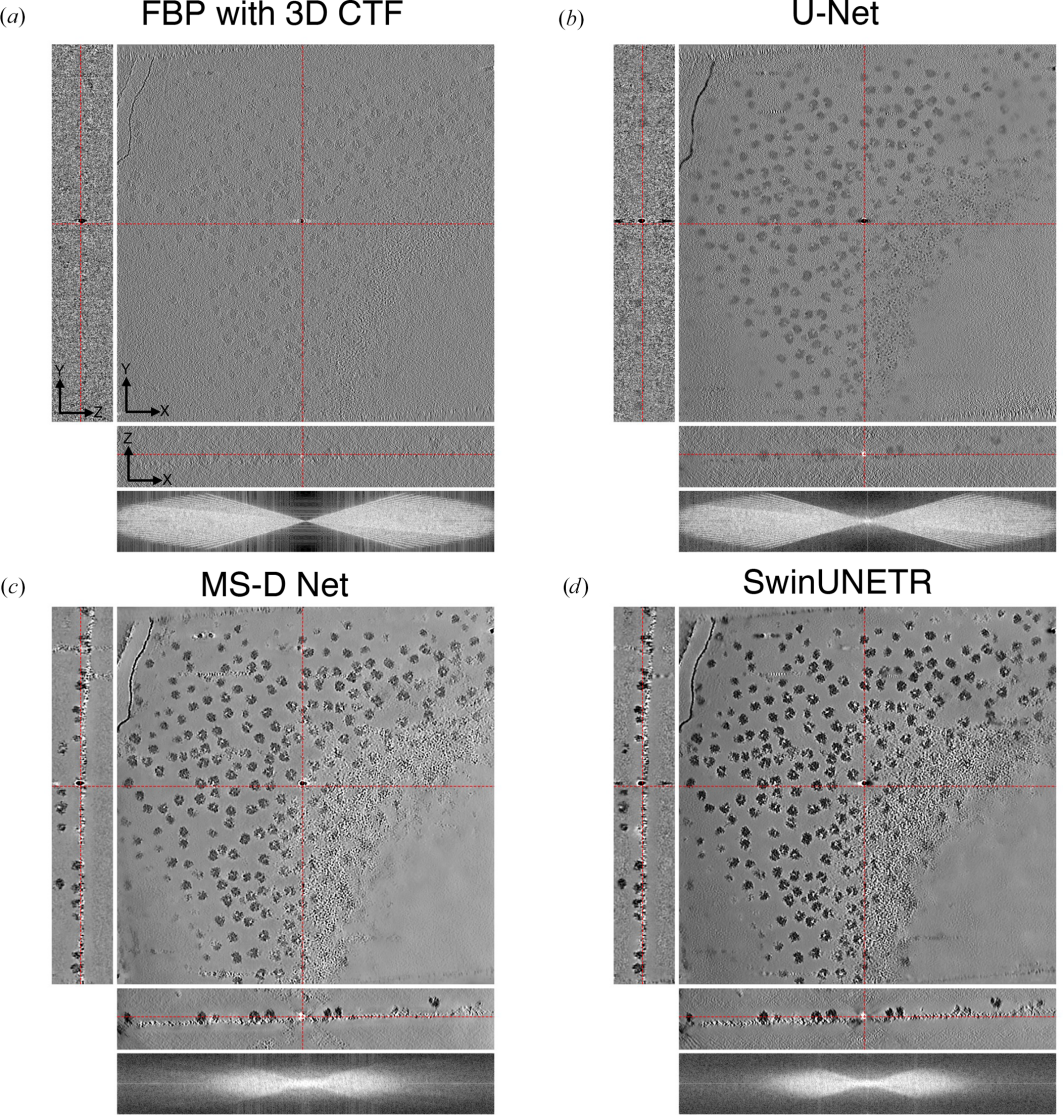
Visual comparison of (*a*) a raw tomogram reconstructed with FBP and 3D CTF correction and tomograms denoised by a trained *DeepDeWedge* model based on (*b*) U-Net, (*c*) MS-D Net and (*d*) SwinUNETR.

**Figure 4:**
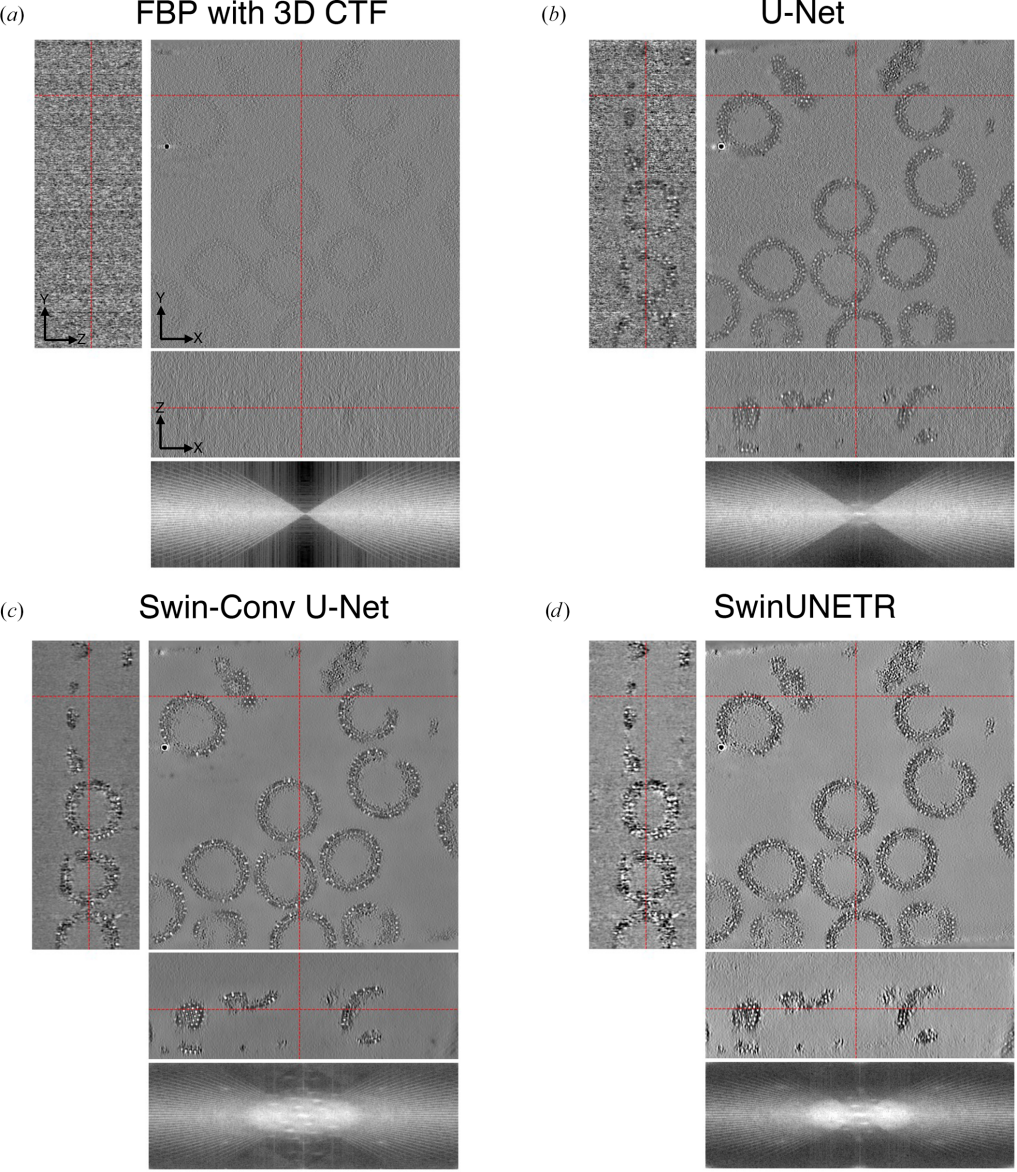
Visual comparison of (*a*) a raw tomogram reconstructed with FBP and 3D CTF correction and tomograms denoised by a trained *DeepDeWedge* model based on (*b*) U-Net, (*c*) SCUNet and (*d*) SwinUNETR. Note the Bragg spots in the log-magnitude Fourier space for denoising with vision transformer architectures.

We qualitatively compare the performance of U-Net, MS-D Net, SCUNet and SwinUNETR for *CryoCare* training on INS-1E cells at the 4× binning level (13.64 Å per pixel; Fig. 2 ▸), *DeepDeWedge* training on EMPIAR-10045 (Fig. 3 ▸) at the 4× binning level (8.704 Å per pixel) and *DeepDeWedge* training on EMPIAR-10164 at the 4× binning level (5.4 Å per pixel; Fig. 4 ▸). While *CryoCare* is trained across a single INS-1E tomogram, *DeepDeWedge* has been trained across six tomograms for EMPIAR-10045 and five tomograms for EMPIAR-10164. As implemented in *CryoCare* (Buchholz *et al.*, 2019 ▸), we found the data-augmentation strategy of four random rotations about the tilt axis to be critical to the performance of the U-Net. Thus, we always employ this type of data augmentation when training *CryoCare* models with L_2_ loss. While the MS-D Net appears to most significantly increase contrast and improve interpretability of the *YZ* slice, cellular features generally appear more blurred than with the U-Net or SCUNet. The SCUNet appears to achieve the best balance of contrast enhancement with the preservation of details including small particles and membrane components within the small vesicles in the lower portion of the upper grid hole. A comparison of *DeepDeWedge* architectures for EMPIAR-10045 in Fig. 3 ▸ shows that the MS-D Net and SwinUNETR significantly increase contrast compared with the U-Net at this binning level, which is readily observed by the attenuation of high-frequency details in the *XZ* Fourier space panel. The MS-D Net and SwinUNETR also improve missing-wedge compensation in comparison to the U-Net. We note here that while the denoising performance of the trained U-Net at 4× binning is weak, at 6× binning the U-Net denoising is comparable to that of our proposed architectures, as shown in Supplementary Figs. S2 and S3. Similar improvements for tomograms of immature HIV-1 VLPs are shown in Fig. 4 ▸, where the transformer-based architectures achieve better contrast, evident in the attenuation of high-frequency Fourier features, and compensation of the missing wedge, primarily in the low frequencies. Models trained with SCUNet and SwinUNETR architectures appear to show an improved appearance of Bragg spots (white spots) in the denoised and missing-wedge-corrected volumes compared with the minimal appearance when processed with the U-Net architecture and apparent absence in the unprocessed tomogram. A quantitative comparison of estimated SNR for ribosomes, INS-1E cells and immature HIV-1 VLPs is shown in Table 1 ▸. In all cases, alternative architectures increase the estimated SNR over the *DeepDeWedge* U-Net. For *CryoCare* training on INS-1E cells and *DeepDeWedge* training on purified ribosomes the MS-D Net achieves the highest estimated SNR, while for *DeepDeWedge* on HIV-1 VLPs SwinUNETR achieves the highest SNR.

### 3.2. Fourier shell correlation as a loss function

**Figure 5:**
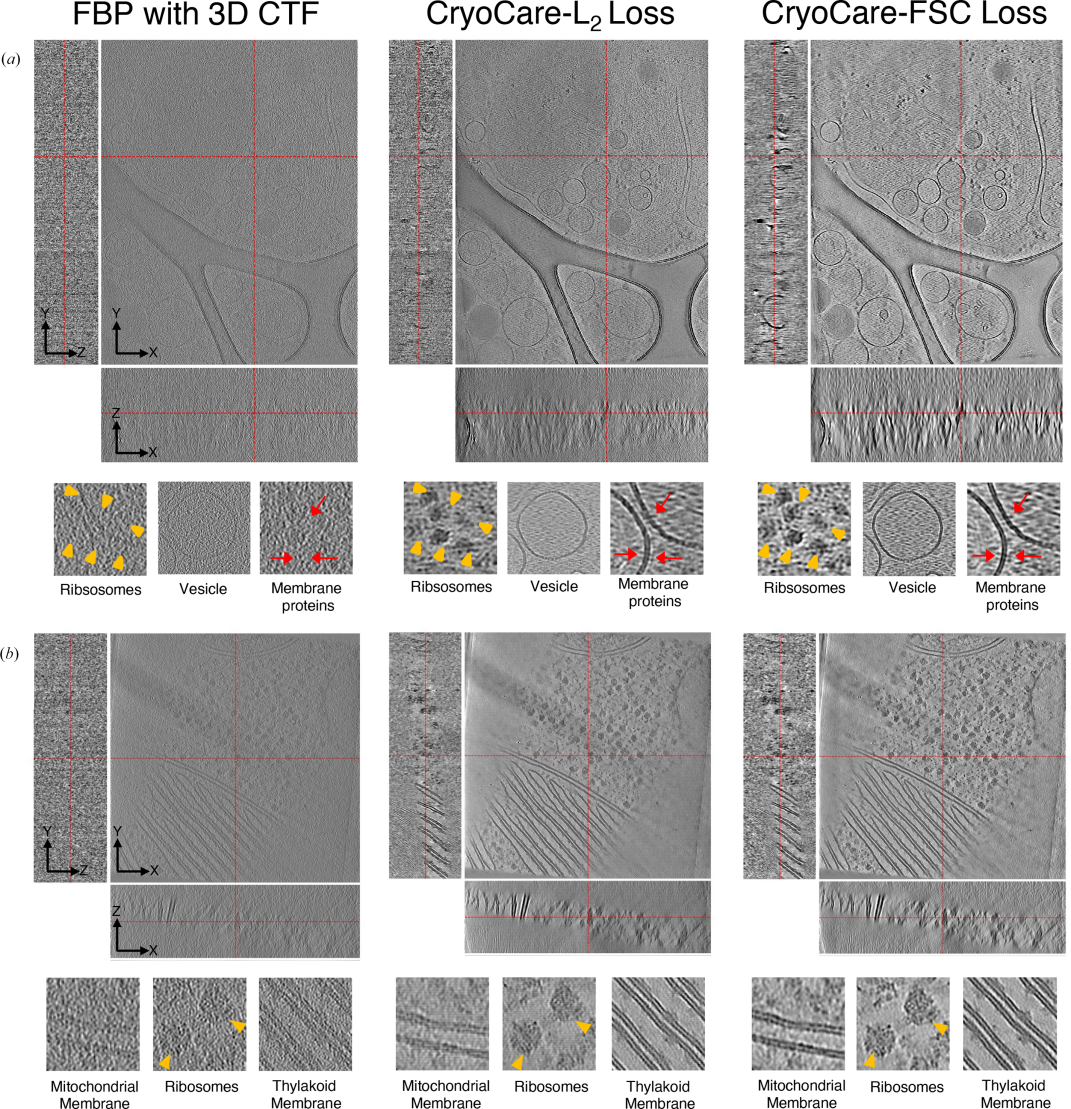
Effectiveness of training *CryoCare* with FSC loss for improving contrast enhancement. A comparison of orthogonal slices of (*a*) INS-1E cells and (*b*) tomogram 24 from EMPIAR-11830 through the raw tomogram and denoised volumes predicted by models trained with L_2_ loss and FSC loss.

While much research in improving denoising has focused on architectures and the generation of training data, there has been considerably less focus on the role played by the training-loss function on the preservation of high-frequency information and the quality of estimated signal. The default training loss for *CryoCare* and *DeepDeWedge* is the L_2_ norm, while *IsoNet* uses the L_1_ norm. The L_2_ norm is known to promote overly smooth images during the training process. A previous study (Bepler *et al.*, 2020 ▸) showed only a marginal difference in performance between training losses based on the L_1_ and L_2_ norms. Here, we propose a loss based on the Fourier shell correlation (FSC) for self-supervised image denoising. Fourier-space supervision losses have been shown to improve the prediction of missing high-frequency content in perceptual image super-resolutions (Fuoli *et al.*, 2021 ▸) and spatiotemporal prediction problems on signal-based data (Yan *et al.*, 2024 ▸). Fourier ring correlation has also been used to train neural networks for image denoising on different noise types (Kaczmar-Michalska *et al.*, 2022 ▸). In particular, a loss function derived from the FSC has been introduced to train a denoiser to enhance the signal in noisy 3D cryo-EM maps that are produced with standard single-particle data-processing pipelines (Agarwal *et al.*, 2024 ▸). FSC is the most commonly used metric for assessing map quality in cryo-EM reconstructions (Saxton & Baumeister, 1982 ▸; Rosenthal & Henderson, 2003 ▸). It measures the normalized cross-correlation between two volumes over corresponding shells in Fourier space,
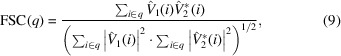
where 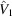 and 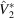 represent the Fourier transform and conjugate Fourier transform, respectively, of the two volumes, *q* is the Fourier shell being considered and the summation is performed over all Fourier voxels *i* that are contained in shell *q*. FSC values lie in the range [−1, 1], with +1 corresponding to perfectly correlated images and −1 corresponding to negatively correlated images. A scalar loss score is calculated by integrating (using discrete quadrature) over all Fourier shells up to the edge of the Fourier volume,
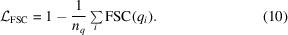
We show a qualitative comparison between predictions from *CryoCare* models trained on a single INS-1E tomogram in Fig. 5 ▸(*a*) and four *C. reinhardtii* tomograms in Fig. 5 ▸(*b*) using the L_2_ and FSC losses. For a fair comparison between the two losses, we used a custom implementation of *CryoCare* training in our forked *DeepDeWedge* repository to train the default *DeepDeWedge* U-Net on 527 subtomogram training pairs extracted by T2T splitting for INS-1E and 467 subtomogram training pairs extracted by F2F splitting for *C. reinhardtii* tomograms. The orthogonal slices through the real-space and zoomed-in sections of ribosomes, membranes and membrane-embedded proteins shown in Fig. 5 ▸ clearly show improved contrast enhancement when the network is trained with the FSC loss compared with the L_2_ loss. The INS-1E tomogram trained with tilt-split tomograms appears to show the FSC loss to primarily increase contrast compared with training with the L_2_ loss, which is best seen in the *YZ* plane. An increase in contrast would reasonably be expected to be accompanied by increased blurriness; however, we observe clearer delineations of vesicle membranes with the FSC loss in the insets of Fig. 5 ▸(*a*), while the ribosome inset primarily demonstrates an increase in contrast. In the *C. reinhardtii* tomogram trained on movie-split data, contrast is similarly improved with the FSC loss; however, the apparent retention of higher frequency details in ribosomes and photosystem II complexes embedded in the thylakoid membrane is more noticeable. In contrast to the INS-1E tomogram, the *YZ* slice of the *C. reinhardtii* tomogram trained with the FSC loss appears to be slightly less interpretable than when trained with the *L*_2_ loss, at least in the visual determination of lamellae thickness. We believe that part of this effect is explained by the differences in the performance of the two losses when training data are generated by T2T or F2F. Splitting the tilt-series images by tilt angle (T2T) decreases the structural similarity between even and odd reconstructions to a greater degree than the movie-split scheme (F2F), which would seem to create a ‘more distant’ N2N mapping to be learned. This increase in mapping ‘distance’ appears to lead to more blurred denoising results, as can be seen in Supplementary Fig. S5, where F2F (movie-split) and T2T (tilt-split) *CryoCare* denoising results on two *C. reinhardtii* tomograms are shown for the L_2_ and FSC losses. These two tomograms appear to show that for the T2T scheme, the FSC loss does appear to blur membrane boundaries to a greater degree than observed in the T2T INS-1E cells presented above. However, in the more statistically principled F2F scheme (Peck *et al.*, 2025 ▸), we observe the FSC loss to achieve a more evenly distributed set of Fourier amplitudes which extend out to higher spatial frequencies than the L_2_ loss, in addition to the qualitatively improved denoising performance discussed above.

For INS-1E tomograms we estimated the SNR for raw (−31.43), *CryoCare* training with L_2_ loss (−12.36) and *CryoCare* training with FSC loss (−21.09). *CryoCare* with L_2_ loss training achieves a higher estimated SNR, further suggesting that the FSC loss retains a greater amount of medium-frequency signal. We note that for INS-1E cells, SNR was calculated by selecting only ribosome particles as ‘signal’ for our simple approximation of contrast. The crowdedness of the FIB-milling cellular tomograms for *C. reinhardtii* prevented a reasonable choice of a background region for SNR estimation for this data set.

We note here that we also trained an *IsoNet* model to compensate for the missing-wedge frequencies (without denoising) with FSC loss self-supervision. As shown in Supplementary Fig. S4, the FSC-trained *IsoNet* model is not as effective in estimating the missing-wedge frequencies when compared with the default L_1_ loss-trained model. This is because for *IsoNet*’s pure missing-wedge estimation task, a frequency-space loss amounts to a challenging image-inpainting task, where the network attempts to predict a region of target Fourier coefficients from a missing region of input Fourier coefficients.

#### 3.2.1. Quantifying preservation of information through subtomogram averaging

**Figure 6:**
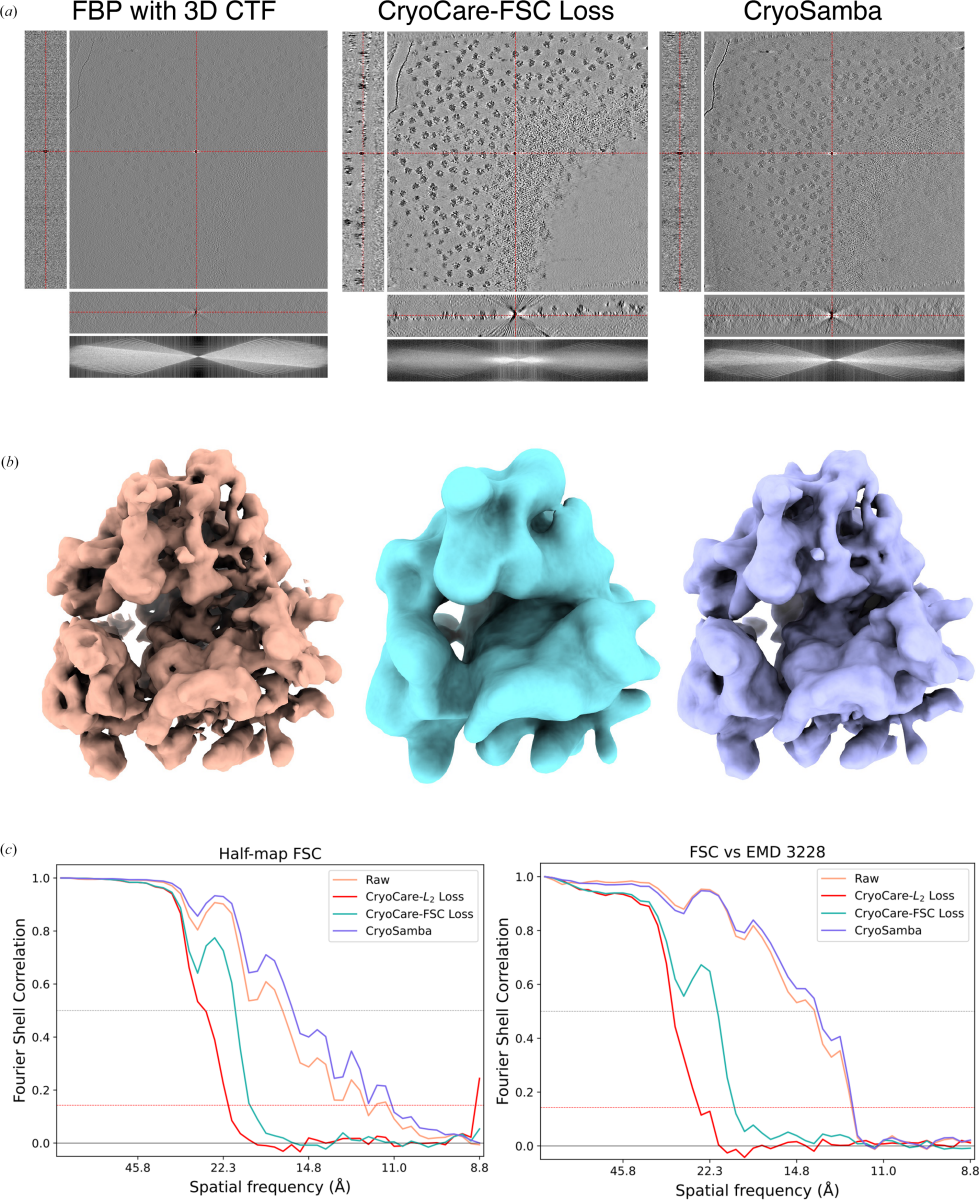
STA of ribosome particles from the EMPIAR-10045 data set. (*a*) Orthogonal slices from real space for raw, *CryoCare*–FSC denoised and *CryoSamba* denoised tomograms. (*b*) STA reconstructions for ribosomes from raw and denoised tomograms. (*c*) Half-map FSC and FSC versus EMDB entry EMD-3228 for STA from raw and denoised tomograms.

To quantify the extent to which denoising with L_2_ and FSC loss attenuates high-resolution structural details, we employed a basic STA procedure. We first used 3D TM as implemented in *GAPSTOP* to extract ribosome particles across five tomograms from EMPIAR-10045 followed by STA with *STOPGAP* (see Sections 2.2.2 and 2.2.3 for details). This simple STA procedure on the raw data resulted in FSC resolutions of 16.4 and 11.6 Å at the 0.5 and 0.143 FSC thresholds, respectively, at the 2× binning (4.35 Å per pixel) level. Next, we used the particle-orientation parameters determined from our final STA iteration to extract and average particles from 2× binned denoised volumes predicted by (i) *CryoCare* trained with FSC loss, (ii) *CryoCare* trained with L_2_ loss and (iii) *CryoSamba*. Slices through the raw and denoised tomograms in Fig. 6 ▸(*a*) shows that *CryoCare*–FSC and *CryoSamba* have improved the SNR compared with the raw tomogram. However, a qualitative comparison of the Fourier space (log-magnitude scale) shows that *CryoSamba* preserves more high-frequency information than *CryoCare* trained with FSC loss, albeit at the cost of decreased contrast enhancement. This is further evident from the differences in the level of structural detail of ribosome densities (Fig. 6 ▸*b*) and their corresponding half-map FSC shown in Fig. 6 ▸(*c*). A comparison of ribosome half-map FSC (Fig. 6 ▸*c*) for raw data, *CryoCare* models trained with L_2_ loss (red) and FSC loss (cyan) and *CryoSamba* (purple) shows that while the *CryoCare* model trained with FSC loss is able to preserve more mid-resolution data compared with that trained with L_2_ loss, *CryoSamba*’s strategy for contrast enhancement by averaging ‘motion-compensated’ nearby planes better preserves high-resolution structural information. In fact, it is surprising that the *CryoSamba* map has similar half-map resolution and very close correspondence in structural features to the map derived from raw tomograms. We also note that despite Fig. 6 ▸(*c*) seemingly showing that *CryoSamba* achieves higher 0.5 and 0.143 FSC resolutions than the raw data, the densities in Fig. 6 ▸(*b*) prove this to not be the case. First demonstrated in Fig. 6 of the *CryoSamba* publication (Costa-Filho *et al.*, 2025 ▸), half-map FSCs of denoised volumes are often spurious, which is why we additionally include the FSC versus EMDB entry EMD-3228 in Fig. 6 ▸(*c*). Since both *CryoCare* and *CryoSamba* do not predict frequencies in the missing wedge, we also trained separate *IsoNet* models on 2× binned *CryoCare*–FSC loss and *CryoSamba* denoised volumes and repeated a similar STA procedure on missing-wedge compensated volumes. Unsurprisingly, these STA results show that missing-wedge estimation has no effect on STA resolution (see Supplementary Fig. S6), as the predicted missing-wedge coefficients do not provide additional independent structural information, but are contextually informed estimations. We conducted a similar analysis for HIV-1 VLP capids with the F2F splitting scheme, where we used raw STA parameters to extract and average particles from denoised volumes. We observe similar qualitative and quantitative results with *CryoCare* with L_2_ and FSC losses and with *CryoSamba* (Supplementary Fig. S7).

#### 3.2.2. FSC loss improves membrane segmentation

**Figure 7:**
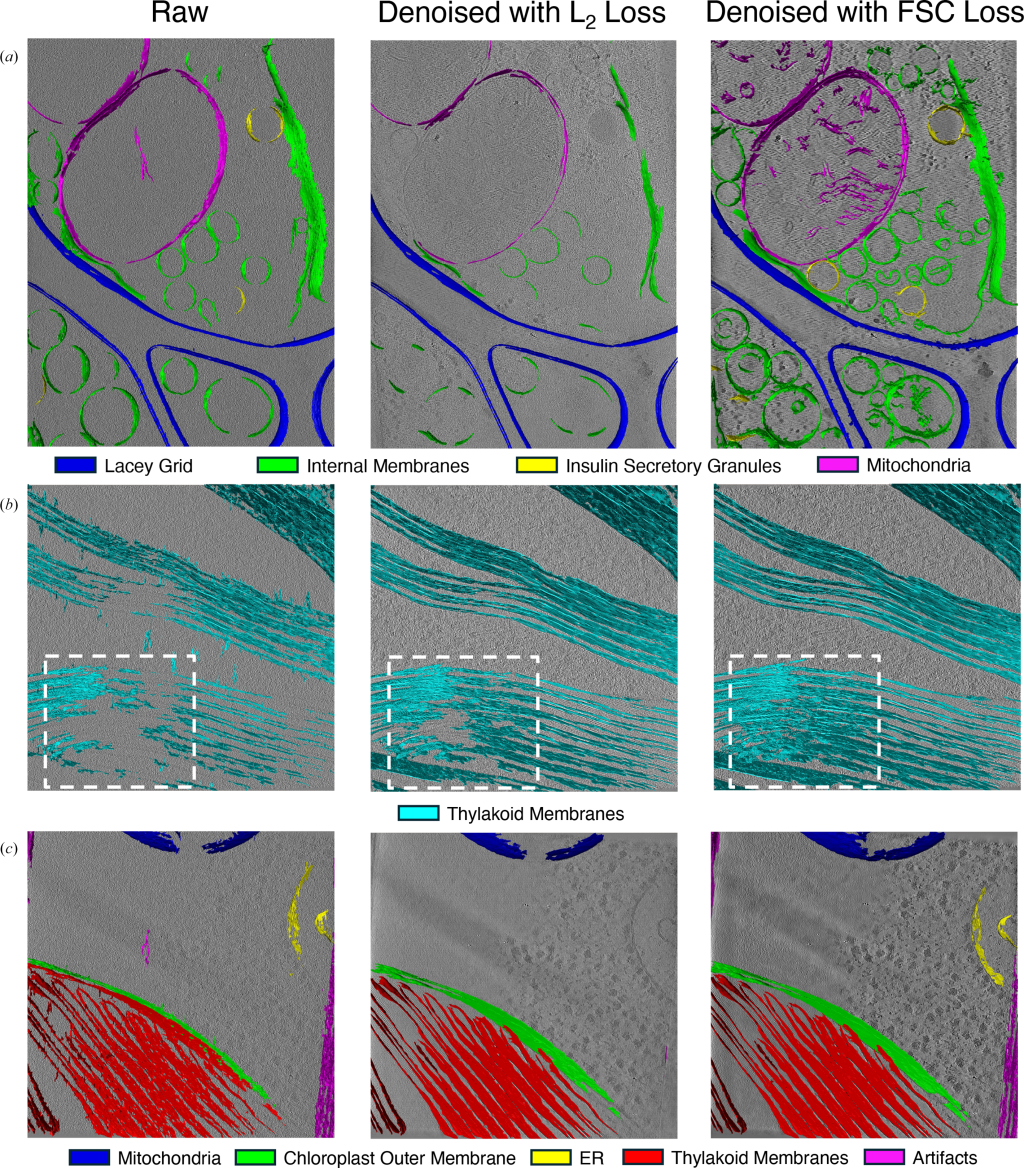
Visual comparison of the effect of L_2_ and FSC loss functions on membrane segmentation using *TomoSegMemTV* in (*a*) non-FIB-milled INS-1E cells and (*b*, *c*) FIB-milled *C. reinhardtii* lamellae: (*b*) tomogram 16 FIB-milled to ∼250 nm and (*c*) tomogram 24 FIB-milled to ∼95 nm.

Segmentation of subcellular features in tomograms is significantly aided by improvements in image contrast (Grotjahn, 2025 ▸). Although U-Net-based cryo-ET segmentation software such as *MemBrain-Seg* (Lamm *et al.*, 2024 ▸) can efficiently segment lipid membranes, because we aimed to compare different architectures and evaluate the effects of contrast enhancement, we instead tested the impact of different loss functions on segmentation performance using *TomoSegMemTV* (Martinez-Sanchez *et al.*, 2014 ▸). *TomoSeg­MemTV* was used to automatically segment lipid membranes in INS-1E cells (Fig. 7 ▸*a*) and FIB-milled *C. reinhardtii* data sets (Figs. 7 ▸*b* and 7 ▸*c*). Interestingly, for the INS-1E data set, we observed that *CryoCare* denoising using an L_2_ loss function resulted in significantly less accurate lipid–membrane segmentation (Fig. 7 ▸*a*, middle) compared with the raw tomogram (Fig. 7 ▸*a*, left). In contrast, introduction of the FSC-based loss function proved more beneficial, enabling identification not only of the membranes visible in the raw tomogram but also additional structures, including other insulin-secretory granules (ISGs) and mitochondrial cristae (Fig. 7 ▸*a*, right). Segmentation performance on *C. reinhardtii* tomograms showed less noticeable differences between the raw, *CryoCare*–L_2_ loss and *CryoCare*–FSC loss tomograms. This is likely to be due to the high quality of the raw tomograms and the previously discussed performance of the FSC loss with T2T and F2F data-splitting schemes (Supplementary Fig. S5). The apparently improved retention of higher frequency Fourier coefficients with FSC loss training on F2F split data does not appear to significantly improve segmentation performance over training with the L_2_ loss for *C. reinhardtii* tomograms. As estimated in the CZI data portal (Ermel *et al.*, 2024 ▸), tomograms 16 and 24 (Figs. 7 ▸*b* and 7 ▸*c*, respectively) greatly differ in their lamellae thickness: tomogram 16 is approximately 250 nm thick, while tomogram 24 is approximately 95 nm thick. This difference in thickness may partly explain why denoising does not significantly improve the performance of segmentation for the thinly milled tomogram 24; the raw tomogram appears to already have sufficient contrast for high-quality segmentation. Generally, Fig. 7 ▸ demonstrates that tomograms denoised with the FSC loss consistently display the most thorough membrane segmentation of INS-1E and *C. reinhardtii* tomograms.

### 3.3. Effect of denoising on particle localization

Downstream analyses of cryo-ET tomograms involve the identification and localization of particles of interest embedded in the cellular matrix through TM (Wan *et al.*, 2024 ▸; Cruz-León *et al.*, 2024 ▸) or deep-learning-based (de Teresa-Trueba *et al.*, 2023 ▸; Liu *et al.*, 2024 ▸) particle picking and subsequent structural analysis through STA and classification. Factors such as low SNRs, significant anisotropy due to the missing wedge (Förster *et al.*, 2008 ▸; Bartesaghi *et al.*, 2008 ▸), incomplete angular sampling between tilts (Majtner *et al.*, 2025 ▸) and crowded cellular environments negatively impact the quality of particle picking, leading to high false-positive rates. While contrast enhancement by denoising improves the visual quality and membrane segmentation (as shown above), an important question to be considered here is how the trade-off between increased SNR and the loss of high-frequency information affects 3D TM and the stability and accuracy of STA parameters.

**Figure 8:**
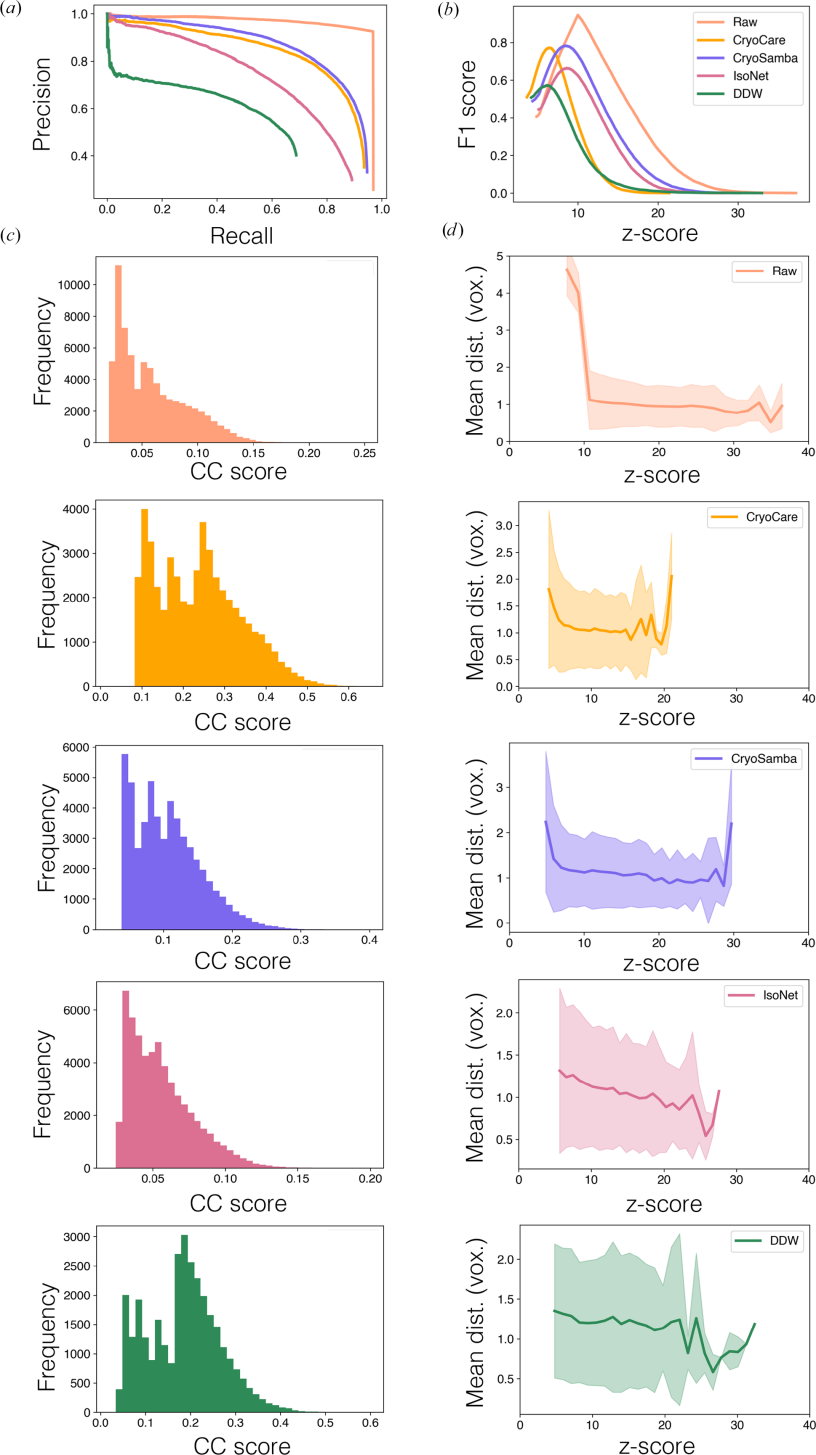
3D TM on raw and denoised volumes for VLPs for five tomograms from EMPIAR-10164. (*a*) Precision–recall curve, (*b*) F1 score, (*c*) histogram of CC scores from peaks selected by thresholding at the 99.5th percentile and (*d*) mean distance (in voxels) between ground-truth particle positions and particles localized by 3D TM. We compare 3D TM for raw tomograms (salmon) and tomograms denoised with *CryoCare* (orange), *CryoSamba* (purple), *IsoNet* (pink) and *DeepDeWedge* (green).

To quantify the impact of denoising on particle detection, we performed 3D TM as implemented in *GAPSTOP* (Cruz-León *et al.*, 2024 ▸). We used a subset of five tilt series from EMPIAR-10164 and performed TM to localize HIV-1 VLP capsids in 4× binned raw tomograms reconstructed with FBP and 3D CTF correction and tomograms denoised by (i) *CryoCare*, (ii) *CryoSamba*, (iii) *IsoNet* and (iv) *DeepDeWedge* models, all trained with default settings. To curate a list of high-quality VLPs to be used as ground truth, we performed STA on raw data at the 2× binning level after initial template matching at the 4× binning level, as detailed in the section on the STA protocol (Section 2.2.3). *STOPGAP* reported this final *B*-factor-sharpened density to reach 6.6 Å resolution at the 0.5 FSC threshold with a cylindrical mask to isolate the central capsid. We computed precision, recall, F1 score and the mean distance between true-positive TM peaks and the corresponding STA positions to assess the impact of different denoising models on particle localization. A comparison of the results for 3D TM on HIV-1 VLP capsids for the raw and different denoising methods is shown in Fig. 8 ▸. The precision–recall curves show that TM on raw data (with PR-AUC = 0.93 and peak F1 = 0.94) consistently achieves higher precision at a given recall across all recall values with lower CC scores. Among the different denoising methods, *CryoSamba* (PR-AUC = 0.82 and peak F1 = 0.78) and *CryoCare* (PR-AUC = 0.79 and peak F1 = 0.77) show comparable performance, while *IsoNet* (PR-AUC = 0.68 and peak F1 = 0.66) and *Deep­DeWedge* (PR-AUC = 0.44 and peak F1 = 0.57) perform substantially worse. Histograms of TM cross-correlation (CC) scores for selected particles (Fig. 8 ▸*c*) show that denoising increases the mean CC of the TM-score distributions while generally broadening the distribution (increasing variance). Consistent with this observation, the F1 score as a function of *Z*-score (Fig. 8 ▸*b*) has a well defined peak at higher *Z*-scores for raw tomograms, whereas denoised tomograms have flatter F1 score profiles, indicating reduced sensitivity to threshold selection. Moreover, Fig. 8 ▸(*d*) shows that despite higher CC scores, the mean distance between ground-truth and template-matched particles does not improve for denoised volumes, and in some cases exhibits increased variance.

**Figure 9:**
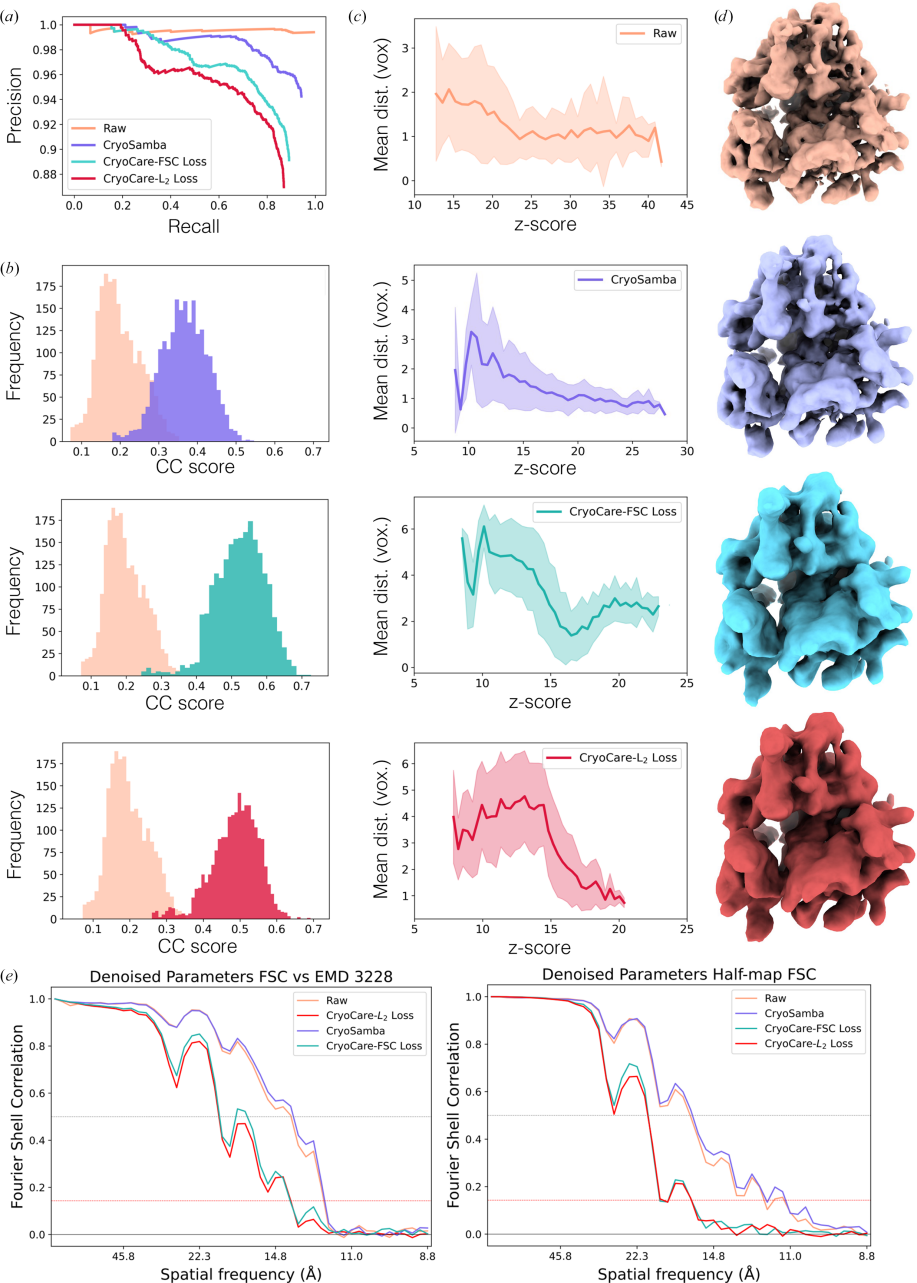
TM and STA results on raw and denoised tomograms. (*a*) Precision–recall curves for TM positions on denoised tomograms, where the finalized raw STA positions are used as ground truth. (*b*) Particle-score histograms compared with the noisy volume. (*c*) Mean distance in pixels between TM peaks and final positions of the raw STA parameters. (*d*) 3D densities and (*e*) FSC versus EMDB entry EMD-3228 and half-map FSC curves when using orientation parameters from denoised tomograms to extract and average subtomograms from the raw tomograms.

Additionally, to investigate the stability and accuracy of the alignment parameters obtained from STA on denoised tomograms, we applied the same 3D TM and STA protocol described in Section 3.2.1 to the same EMPIAR-10045 tomograms denoised with *CryoCare*–L_2_ loss, *CryoCare*–FSC loss and *CryoSamba*. Note that *DeepDeWedge* fails to be effective at denoising or predicting missing-wedge frequencies at 2× binning, and is therefore excluded from this analysis. We used the final alignment parameters from each of the STA runs on the denoised tomograms at 2× binning to extract and average particles from the raw 2× binned tomogram. Fig. 9 ▸ shows results for TM detection and STA orientation stability and accuracy on 80S ribosomes from EMPIAR-10045. As in the HIV-1 VLP analysis, precision–recall curves and mean distance measurements show that TM on raw tomograms outperforms that on denoised tomograms. Not too surprisingly, the effect persists when denoised volumes are used to obtain alignment parameters for STA, as evident from the detail in STA densities (extracted from raw tomograms) in Fig. 9 ▸(*d*) and the corresponding FSC curves in Fig. 9 ▸(*e*). Averages obtained using raw and *CryoSamba* STA parameters reached approximately 16.5 and 11.3 Å at the 0.5 and 0.143 FSC thresholds, respectively, while averages obtained using *CryoCare*–FSC and *CryoCare*–L_2_ STA parameters both reached approximately 21.2 and 19.3 Å at the 0.5 and 0.143 FSC thresholds, respectively. This further confirms our observations from Section 3.2.1 that *CryoSamba* preserves a greater fraction of the high-frequency signal that is needed for accurate alignment parameters. These results demonstrate that although denoising inflates cross-correlation scores, it degrades the discriminative power of TM for separating true- and false-particle detection, likely due to the loss of high-frequency structural information critical for accurate particle localization.

## 4. Discussion

Cryo-ET is a rapidly emerging technique that enables the 3D visualization of complex biological structures in near-native cellular environments. However, constraints of the imaging system and dose limits to avoid radiation damage result in 3D tomograms with low SNR and missing-wedge artifacts, which make direct inspection with 3D TM, STA and segmentation challenging. Recently, several deep-learning methods have been proposed to enhance the contrast and in some cases jointly reduce reconstruction artifacts. Here, we have presented a systematic analysis of these state-of-the-art methods and proposed improvements in neural network architectures and training objectives to improve the performance of these methods. In particular, we have shown that MS-D Net is a relatively low-training-parameter architecture that is useful in low-data regimes, while attention-enabled vision transformer-based networks have the potential to greatly improve denoising by understanding both local and global contexts, although these methods remain fundamentally limited by the training-data volume. These architectures offer a robust alternative to the standard U-Net and show better contrast enhancement at lower data-binning levels. While the performances of SwinUNETR and SCUNet were often comparable, we did find SwinUNETR more prone to overfitting, making SCUNet a better choice. We have also introduced a training objective based on Fourier shell correlation that helps to preserve mid-frequency signals compared with a standard L_2_ loss function. Our analysis of STA of 80S ribosomes and HIV-1 VLP capsids on tomograms denoised with *CryoCare* models trained with L_2_ and FSC loss functions show that FSC loss achieves higher correlations with high-resolution deposited structures, proving that the FSC loss retains a greater degree of relevant structural information.

Future work on improving the interpretability of cryo-ET reconstructions will focus on methods for jointly reducing missing-wedge artifacts and enhancing contrast. Although *DeepDeWedge* has proven effective at this task for highly binned tomograms when smoothness and high contrast are paramount, we found it to struggle on tomograms with smaller pixel sizes. It is possible that the inherent expressiveness of convolutional kernels are best used for one task at a time, and that convolutional filters learned on both the anisotropy and denoising task may be inherently less effective than those trained on a single task. At the time of revising this manuscript, the authors of *IsoNet* released a variant (Liu *et al.*, 2025 ▸) that takes a step in this direction. Following the implementation of the FSC loss, we did attempt to incorporate the FSC into the masked-loss training scheme of *DeepDeWedge*. As mentioned in Section 3.2, this comes with difficulties unique to Fourier space supervision, and although we did observe acceptable denoising, missing-wedge estimation was not as effective. Not explored in this work are blind-spot denoising models, which have been shown to achieve lower denoising performance than paired schemes in the literature (Wang *et al.*, 2022 ▸), but can begin with twice the signal in cryo-ET by eliminating the need to split data into two reconstructions. Implementation of a blind spot into *IsoNet* could allow denoising within the state-of-the-art method for reducing anisotropy in cryo-ET reconstructions.

A primary goal of this study was to understand the practical limits and utility of self-supervised restoration in biological cryo-ET. Beyond qualitative visualization, we explored the downstream tasks of segmentation, particle localization with 3D TM and 3D structure determination with STA. Our results show that while denoising improves visual interpretability and gives more continuous and complete membrane segmentation, it does not enhance the statistical fidelity required for quantitative particle detection, localization and alignment. Besides the loss of high-frequency information, this degradation in performance could be due to the fact that particle-picking and refinement software assume noise distributions typical of raw data. Another concern when denoising with deep-learning methods is the introduction of hallucinations that lead to distortions in the denoised tomograms. This could be performed by uncertainty-aware denoising based on ensemble methods or probabilistic neural networks (Kohl *et al.*, 2018 ▸) which output prediction distribution statistics along with the denoised output. Denoised representations may further prove useful for post-processing tasks (not considered in the current study) that rely on human or machine interpretation rather than precise localization, such as manual annotation, the exploration of cellular context or refinement of tomographic tilt-series alignment. Lastly, a promising future direction is the development of joint denoising and localization and alignment networks (Huang *et al.*, 2024 ▸) that are trained by optimizing a composite loss function consisting of task-aware loss terms and regularized by frequency-preserving constraints.

## 5. Related literature

The following reference is cited in the supporting information for this article: Cardoso *et al.* (2022 ▸).

## Supplementary Material

Neural network architectures and Supplementary Figures. DOI: 10.1107/S2059798326001166/sor5005sup1.pdf

## Figures and Tables

**Table 1:** Quantitative comparison of the denoising performance of different architectures with estimated signal-to-noise ratio

	Raw	U-Net	MS-D Net	SwinUNETR	SCUNet
*CryoCare* on INS-1E	−31.43	−12.36	**−7.30**	−11.30	−12.18
*DeepDeWedge* on EMPIAR-10045 ribosomes	−34.89	−24.76	**−10.62**	−12.01	−11.15
*DeepDeWedge* on EMPIAR-10164 HIV-1 VLPs	−35.96	−21.62	−11.87	**−8.57**	−11.71

## Data Availability

Tilt-series data sets for *S. cerevisiae* 80S ribosomes and HIV-1 VLPs are available in the EMPIAR database under accession Nos. EMPIAR-10045 and EMPIAR-10164, respectively. Data for INS-1E will be deposited in the EMPIAR database upon publication of the related manuscript (A. Deshmukh & A. Archambeau, in preparation). We used the *DeepDeWedge* repository to integrate implementations of new architectures from their respective sources (details are given in the supporting information).
