# Supplementary material for: Deep-learning methods for contrast enhancement and artifact reduction in cryo-electron tomography: a systematic analysis of the state of the art and proposed improvements
Source: Acta Crystallogr D Struct Biol. 2026 Feb 19;82(Pt 3):168–86. doi: 10.1107/S2059798326001166 (PMC12954857; doi:10.1107/S2059798326001166)
Supplement: Supplementary file 1 [file d-82-00168-sup1.pdf]

Acta Cryst D STRUCTURAL BIOLOGY

Volume 82 (2026)

Supporting information for article:

Deep-learning methods for contrast enhancement and artifact reduction in cryo-electron tomography: a systematic analysis of the state of the art and proposed improvements

Henry N. Jones, Aneesh Deshmukh and Kanupriya Pande

S1 Neural Network Architectures

U-Net The U-Net architecture we used consists of 64 channels and three downsampling layers, giving 27.3 million trainable parameters. This is the default model architecture implemented in the DDW repository (Wiedemann & R.Heckel, 2024). Time to train 100 DDW iterations with U-Net model on 450 input-target pairs is roughly 3 hours 40 minutes.

MS-D Net The MS-D Net architecture has 50 single-channel intermediate layers, and the convolutions in layer i are dilated by $d_i = 1 + (i \bmod 10)$ giving 34,500 trainable parameters. We used the open-source PyTorch implementation of the MS-D Net (Hendriksen, 2020). Time to train 100 DDW iterations with MS-D Net model on 450 input-target pairs is roughly 5 hours.

SwinUNetR The SwinUNetR architecture consists of a U-Net as the backbone with a swin-transformer as an encoder connected to a CNN decoder via skip connections. We used the default model implemented in MONAI (Cardoso et al., 2022; MONAI Consortium, 2025) with drop rate and attention drop rate both set to 0.1, and 15.7 million trainable parameters. Time to train 100 DDW iterations with SwinUNETR model on 450 input-target pairs is roughly 1 hour 40 minutes.

Swin-Conv U-Net SCUNet exploits the Swin-conv (SC) block as the main building block of a U-Net backbone with 32 channels and 3 downsampling layers. Each SC block includes a swin-transformer and a residual convolution block. The model has 4.7 million trainable parameters. We used the SCUNet implementation from EMReady (He et al., 2023). Time to train 100 DDW iterations with SCUNet on 450 input-target pairs is roughly 4 hours 40 minutes.

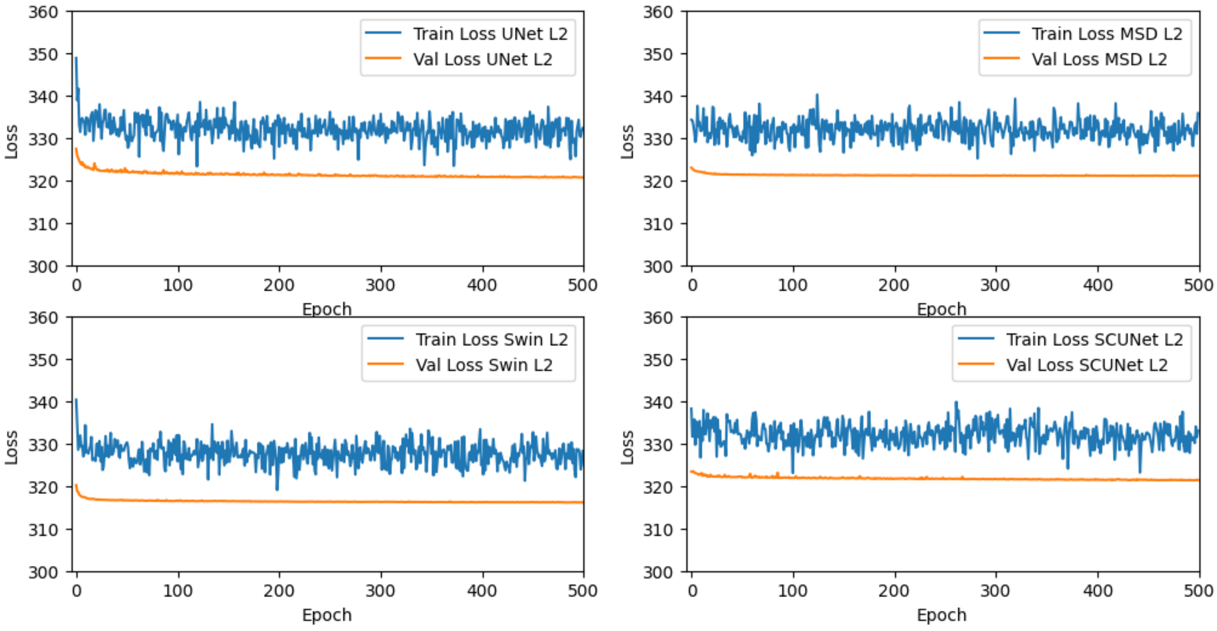

Figure S1: Training and validation losses for training DDW with different model architectures for 450 input-target pairs extracted from 5 tilt-series of EMPIAR-10164.

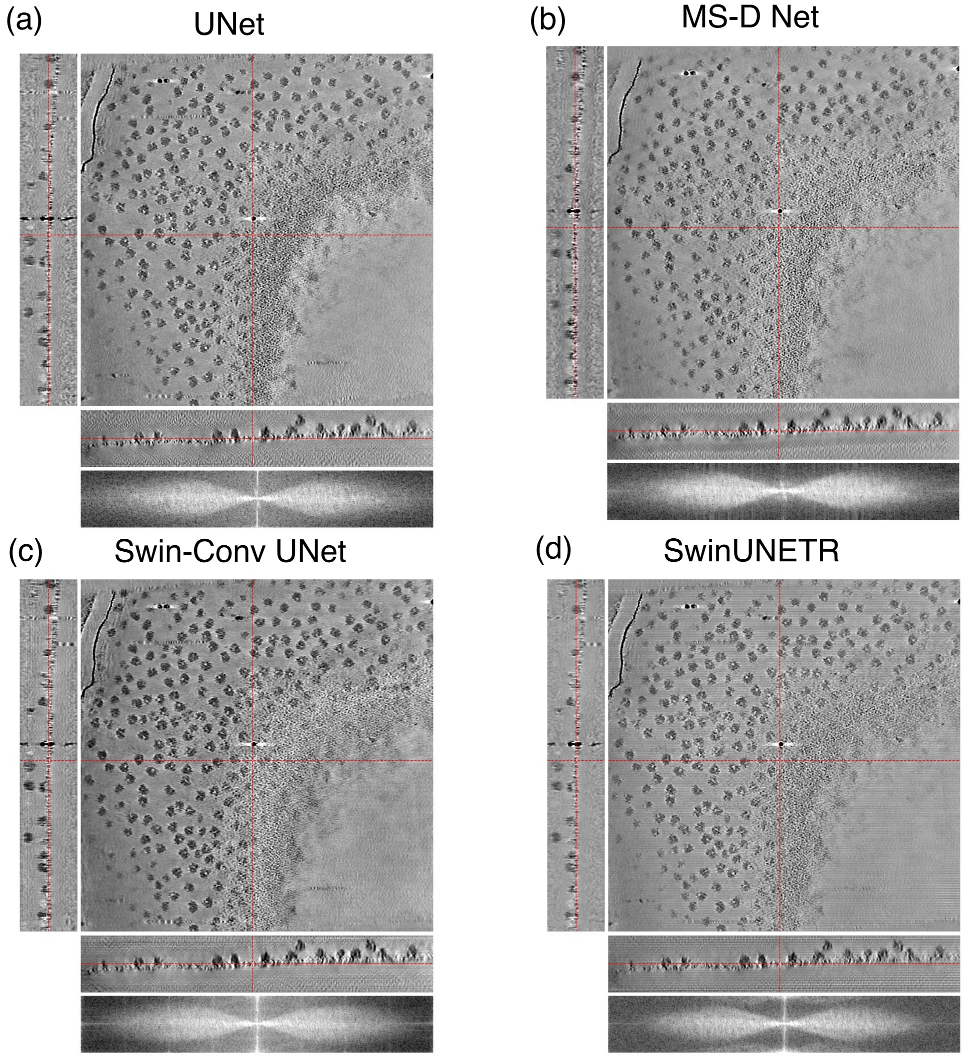

Figure S2: CryoCare trained with different model architectures for EMPIAR-10045 at 6x binning. Visual comparison of orthogonal slices through tomogram 5 for (a) U-Net, (b) MS-D Net, (c) Swin-Conv U-Net, and (d) SwinUNETR.

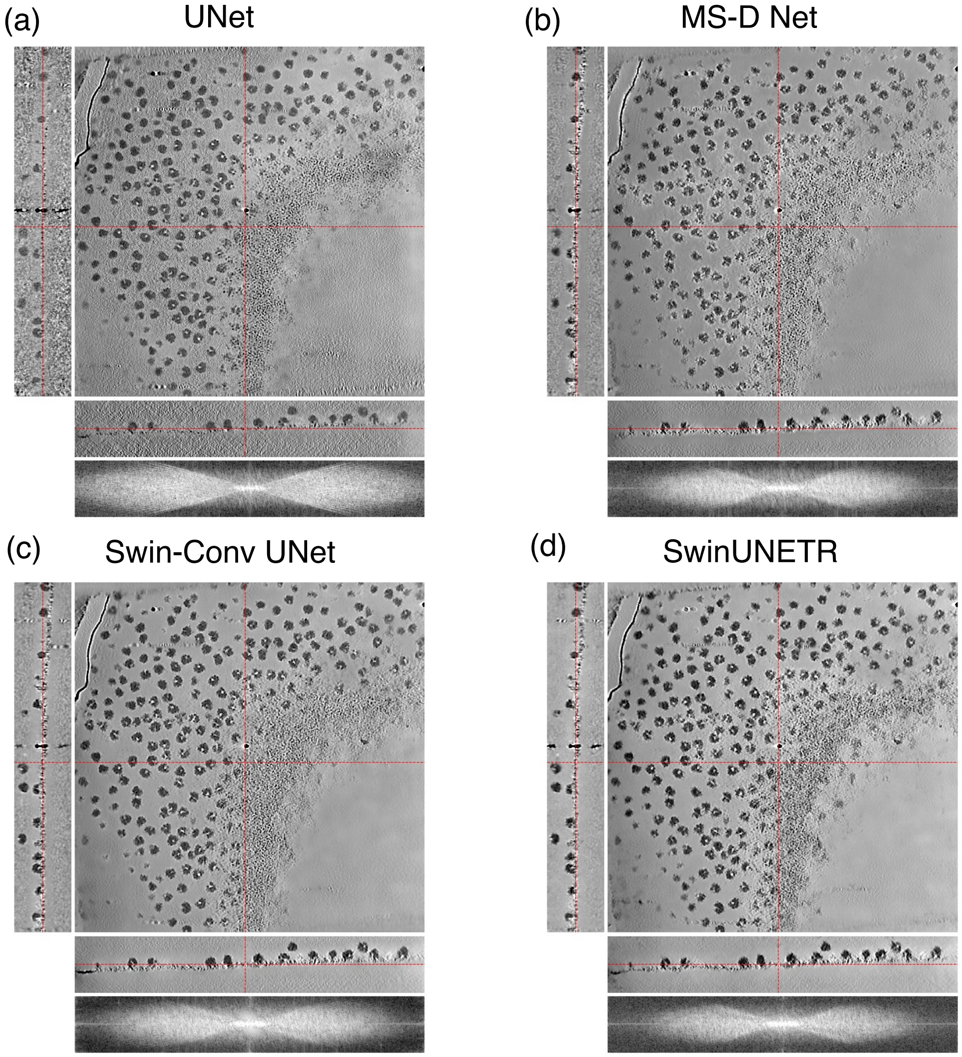

Figure S3: DeepDeWedge trained with different model architectures for EMPIAR-10045 at 6x binning. Visual comparison of orthogonal slices through tomogram 5 for (a) U-Net, (b) MS-D Net, (c) Swin-Conv U-Net, and (d) SwinUNETR.

S2 Training IsoNet with FSC Loss

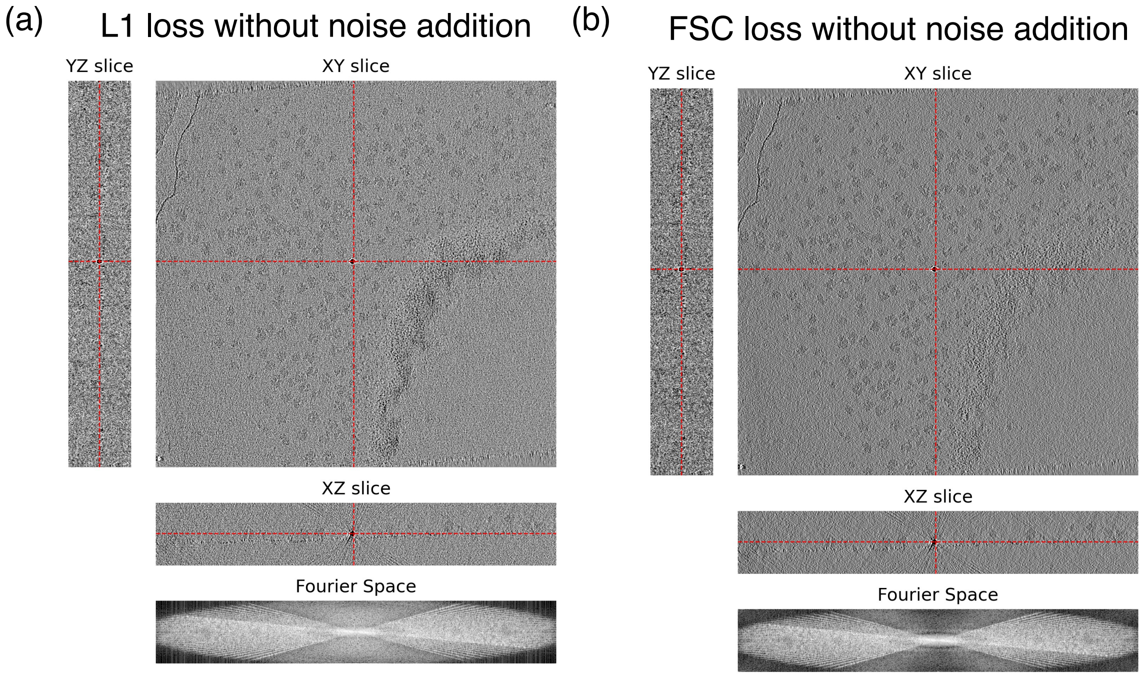

Figure S4: Effectiveness of training IsoNet with FSC loss. Orthogonal slices through the denoised volumes and power-spectrum for IsoNet trained with (left) L1 loss and (right) FSC loss. The training was done without IsoNet's noise addition scheme to evaluate the effect of the FSC loss for missing-wedge compensation.

S3 Comparison of CryoCare denoising with L_2 and FSC losses for training data generated with tilt-split and movie-split

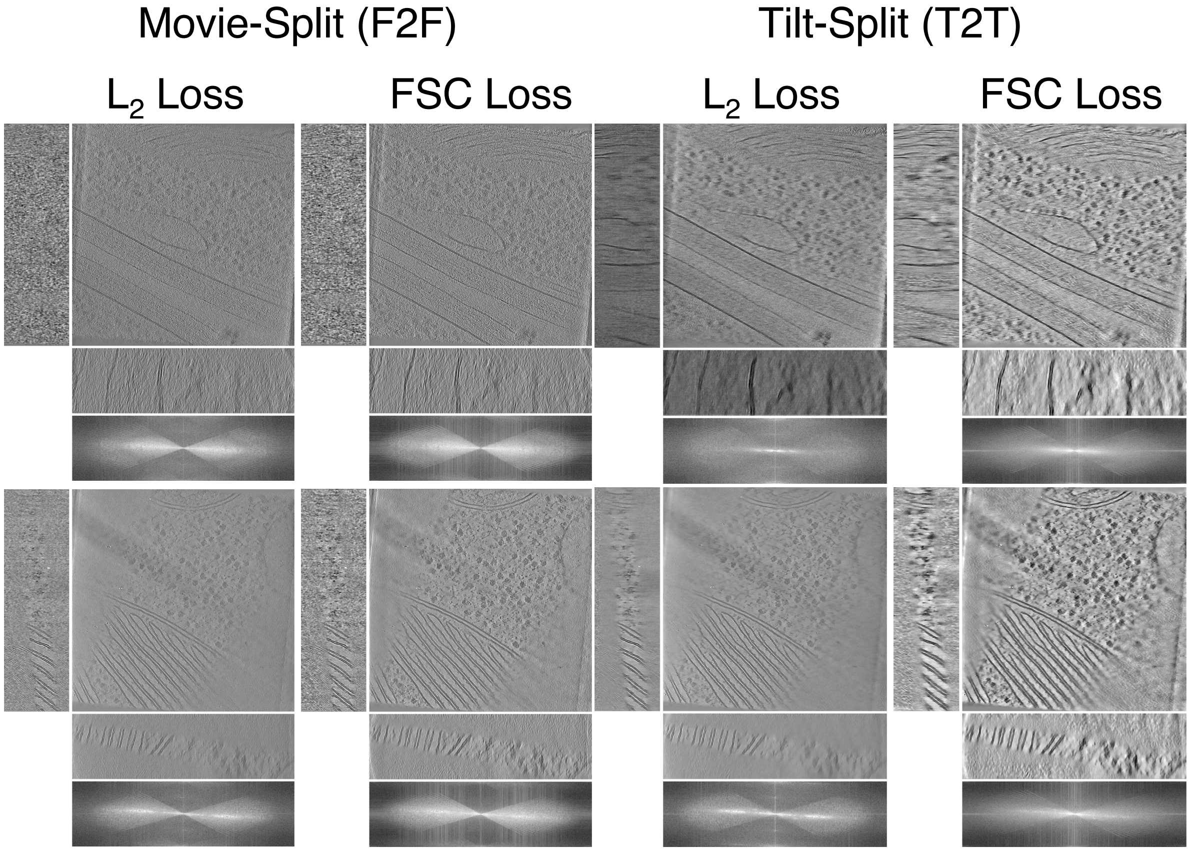

Figure S5: Results for CryoCare trained with the L_2 and FSC losses on movie-split (F2F) and tilt-split (T2T) tomograms 19 (top) and 24 (bottom) of EMPIAR-11830.

S4 STA on denoised volumes for EMPIAR-10045

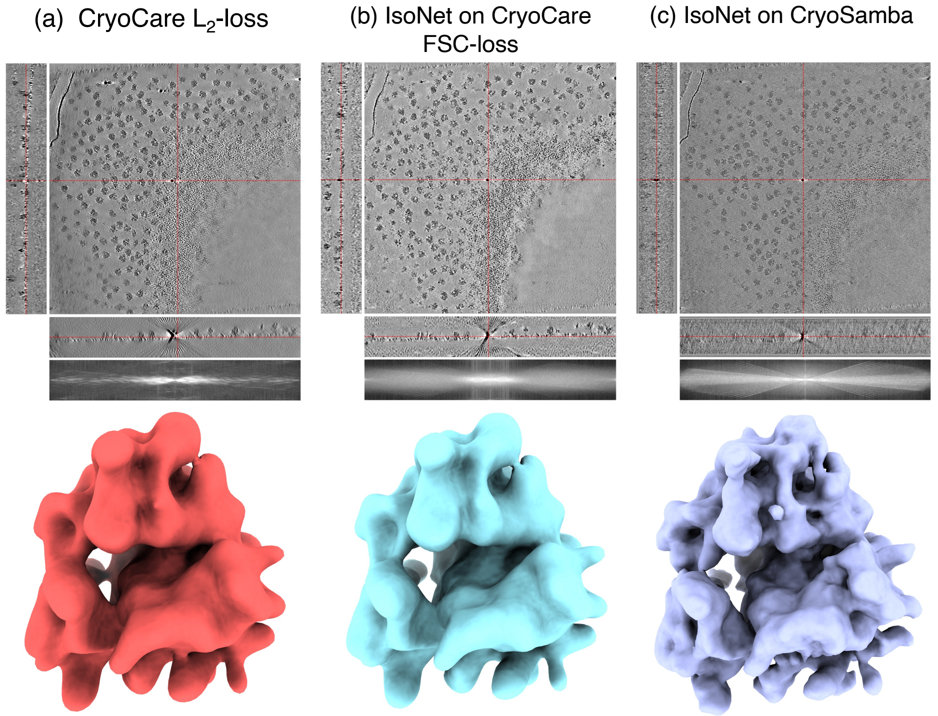

Figure S6: Subtomogram averaging on EMPIAR-10045 for tomograms denoised with (a) CryoCare-L_2 loss, (b) IsoNet trained on volumes denoised with CryoCare-FSC loss and (c) IsoNet trained on volumes denoised with CryoSamba.

S5 STA on denoised volumes for EMPIAR-10164

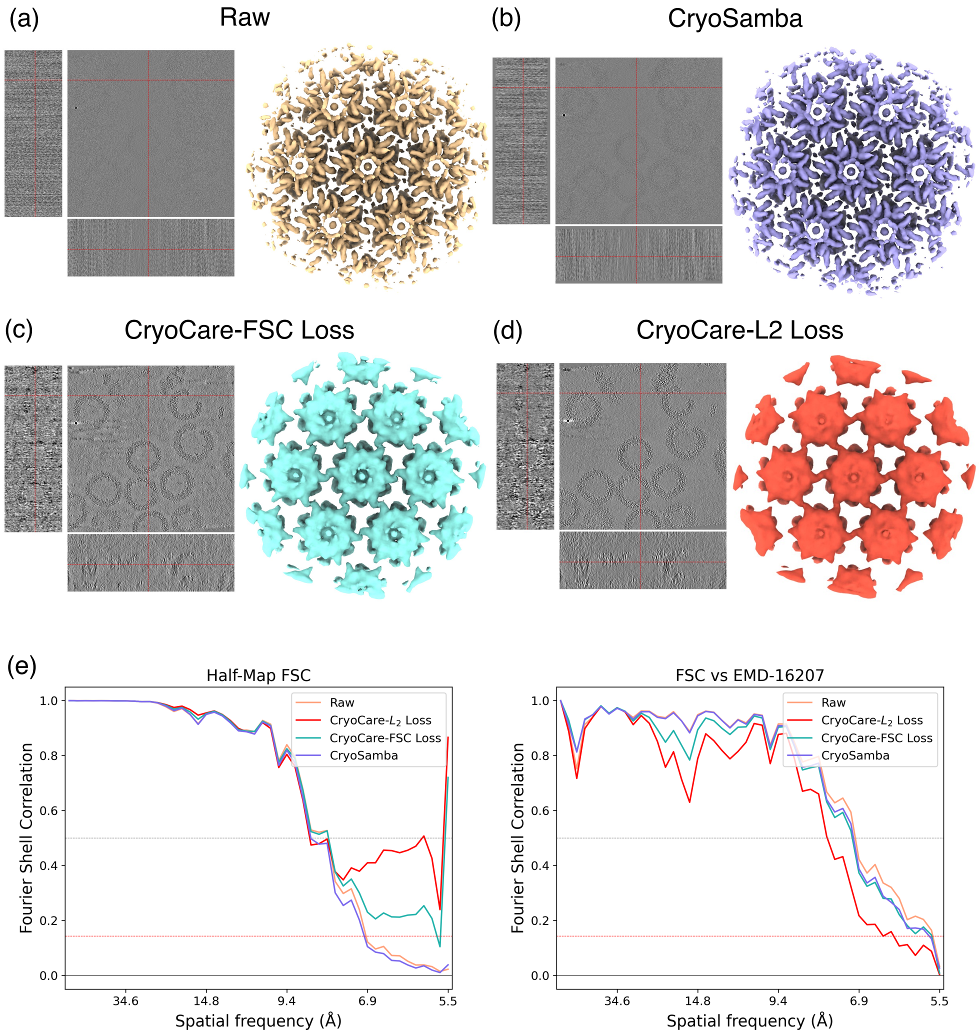

Figure S7: Subtomogram averaging on EMPIAR-10164 for (a) raw tomograms and tomograms denoised with (b) CryoSamba, (c) CryoCare-FSC loss, and (d) CryoCare-L_2 loss. Features on subtomogram averages along with (e) half-map FSC and FSC vs EMD-16207 shows that CryoSamba resolution closely follows that of raw, while CryoCare-FSC loss preserves more medium resolution frequencies as compared to CryoCare-L_2 loss. As demonstrated in Fig. 6 of the CryoSamba publication (Costa-Filho et al., 2025), half-map FSC of denoised volumes are often unreliable, which is why we additionally include the FSC vs EMD-16207.

S6 3D TM for EMPIAR-10045

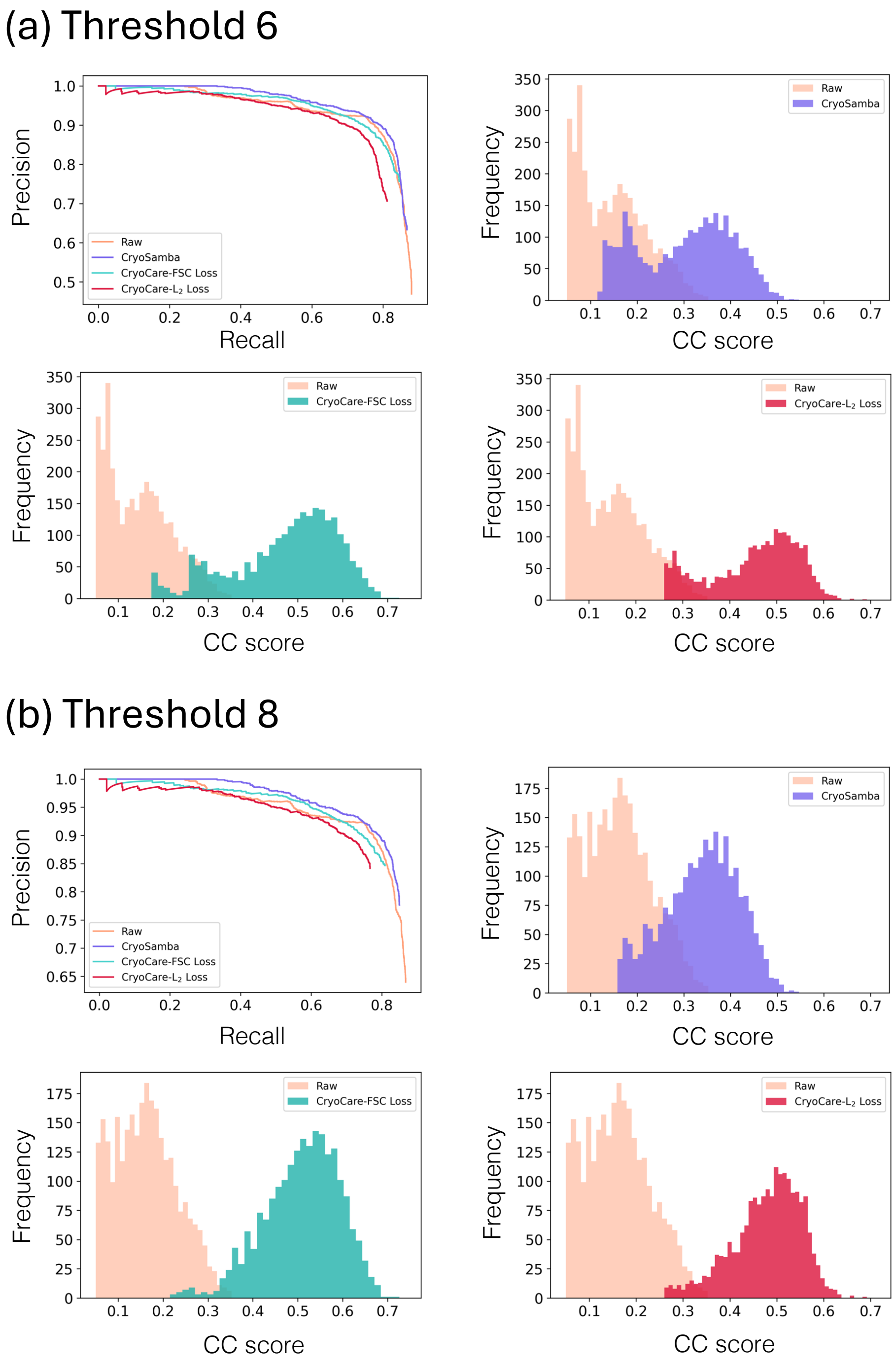

Figure S8: Precision-recall curve and CC score histograms for 3D template-matching on 5 tomograms of EMPIAR-10045 for (a) threshold=6 and (b) threshold=8.

References

Cardoso, M. J., Li, W., Brown, R., Ma, N., Kerfoot, E., Wang, Y., Murrey, B., Myronenko, A., Zhao, C., Yang, D., Nath, V., He, Y., Xu, Z., Hatamizadeh, A., Myronenko, A., Zhu, W., Liu, Y., Zheng, M., Tang, Y., Yang, I., Zephyr, M., Hashemian, B., Alle, S., Darestani, M. Z., Budd, C., Modat, M., Vercauteren, T., Wang, G., Li, Y., Hu, Y., Fu, Y., Gorman, B., Johnson, H., Genereaux, B., Erdal, B. S., Gupta, V., Diaz-Pinto, A., Dourson, A., Maier-Hein, L., Jaeger, P. F., Baumgartner, M., Kalpathy-Cramer, J., Flores, M., Kirby, J., Cooper, L. A., Roth, H. R., Xu, D., Bericat, D., Floca, R., Zhou, S. K., Shuaib, H., Farahani, K., Maier-Hein, K. H., Aylward, S., Dogra, P., Ourselin, S. & Feng, A. (2022). arXiv, **2211.02701**.

He, J., Li, T. & Huang, S.-Y. (2023). http://huanglab.phys.hust.edu.cn/EMReady.

Hendriksen, A. A. (2020). https://github.com/ahendriksen/msd_pytorch.

MONAI Consortium (2025). https://github.com/Project-MONAI/MONAI.

Wiedemann, S. & R.Heckel (2024). https://github.com/MLI-lab/DeepDeWedge.
